# Acupuncture and moxibustion in patients with cancer-related insomnia: A systematic review and network meta-analysis

**DOI:** 10.3389/fpsyt.2023.1108686

**Published:** 2023-02-16

**Authors:** Yangxu Ou, Dezhi Lin, Xixiu Ni, Simeng Li, Kexin Wu, Lu Yuan, Jing Rong, Chengzhi Feng, Junqian Liu, Yang Yu, Xiao Wang, Linjia Wang, Zili Tang, Ling Zhao

**Affiliations:** ^1^Acupuncture and Tuina School, Chengdu University of Traditional Chinese Medicine, Chengdu, China; ^2^Traditional Chinese Medicine School, Hunan University of Traditional Chinese Medicine, Changsha, China; ^3^Hospital of Chengdu University of Traditional Chinese Medicine, Chengdu, China

**Keywords:** cancer-related insomnia, network meta-analysis, systematic review, acupuncture and moxibustion, PSQI

## Abstract

**Objectives:**

Cancer-related insomnia (CRI) is one of the most common and serious symptoms in patients with cancer. Acupuncture and moxibustion have been widely applied in the treatment of CRI. Nevertheless, the comparative efficacy and safety of different acupuncture and moxibustion techniques remain unclear. This study aimed to evaluate and compare the efficacy and safety of different acupuncture and moxibustion techniques in the treatment of CRI.

**Methods:**

Eight medical databases were comprehensively searched for relevant randomized controlled trials (RCTs) as of June 2022. Two independent reviewers assessed the risk of bias and conducted the research selection, data extraction, and quality assessment of the included RCTs. A network meta-analysis (NMA) was performed using frequency models, combining all available direct and indirect evidence from RCTs. The Pittsburgh Sleep Quality Index (PSQI) was set as the primary outcome, and adverse events and effective rates were set as the secondary outcomes. The efficacy rate was calculated as the ratio of patients with insomnia symptom relief to the total number of patients.

**Results:**

Thirty-one RCTs with 3,046 participants were included, including 16 acupuncture- and moxibustion-related therapies. Transcutaneous electrical acupoint stimulation [surface under the cumulative ranking curve (SUCRA) 85.7%] and acupuncture and moxibustion (SUCRA 79.1%) were more effective than Western medicine, routine care, and placebo-sham acupuncture. Furthermore, Western medicine showed significantly better effects than placebo-sham acupuncture. In the NMA, the acupuncture and moxibustion treatments with the best therapeutic effects for CRI were transcutaneous electrical acupoint stimulation (SUCRA 85.7%), acupuncture and moxibustion (SUCRA 79.1%), auricular acupuncture (SUCRA 62.9%), routine care combined with intradermal needling (SUCRA 55.0%), and intradermal needling alone (SUCRA 53.3%). No serious acupuncture- or moxibustion-related adverse events were reported in the included studies.

**Conclusion:**

Acupuncture and moxibustion are effective and relatively safe in treating CRI. The relatively conservative recommended order of acupuncture- and moxibustion-related therapies for CRI is as follows: transcutaneous electrical acupoint stimulation, acupuncture and moxibustion, and auricular acupuncture. However, the methodological quality of the included studies was generally poor, and further high-quality RCTs are needed to strengthen the evidence base.

## 1. Introduction

Cancer is a leading cause of mortality worldwide and its incidence (1). Insomnia is among the most prominent symptoms of cancer, with a prevalence rate of 25–59% (2). Cancer-related insomnia (CRI) is a sleep disorder caused by cancer or its treatment (drugs, surgery, radiotherapy, and chemotherapy), which exposes patients to high risks for physical (e.g., pain, fatigue) and psychological comorbidities (e.g., anxiety and depression), in addition to reduced quality of life (3–5). These challenges reduce compliance with conventional anti-cancer treatments, increase financial burden, and threaten long-term survival (6).

CRI is conventionally treated with pharmacotherapy, including antidepressants, benzodiazepine receptor agonists (BZRAs), melatonin receptor agonists, and antihistamines (7). However, conventional Western medicine (WM) treatment is associated with resistance and side effects, such as drug dependence and residual daytime sedation. In addition, cognitive behavioral therapy (CBT) for CRI is the gold standard, but due to the complexity of treatment steps and relatively high cost, patient compliance and acceptance are relatively low. Therefore, complementary and alternative medicine, including acupuncture- and moxibustion-related therapies, have been increasingly introduced into the management of CRI.

Acupuncture and moxibustion are expected to play a non-pharmacological role in managing CRI with acupoint stimulation as an essential modality, considering their potential to alleviate cancer symptoms and their feasibility without adding burden to patients’ physical and financial situations (8).

In recent years, various acupuncture- and moxibustion-related therapies have been widely applied to the treatment of cancer-related symptoms, including CRI, cancer-related pain, fatigue, and hot flushes (8). Previous studies have shown that auricular acupuncture (AA) (9), scalp acupuncture (SA) (10), electro-acupuncture (EA) (11), and intradermal needling (IN) (12) might be more advantageous in treating CRI than WM, placebo-sham acupuncture (PSA), or routine care (RC). A systematic review (13) published in 2022 found that acupuncture and/or moxibustion have an affirmative effect on the treatment of CRI. Such treatment could be considered an adjuvant alternative to current CRI management. Additionally, another systematic review (14) published in 2022 showed that acupuncture has great potential to manage CRI in cancer patients, while the evidence of true acupuncture, PSA, and WM in treating CRI has not been entirely conclusive. Clinicians have encountered difficulties in selecting the most favorable acupuncture treatment for CRI. Thus, a comprehensive analysis and evaluation of relevant evidence are required.

In this study, a network meta-analysis (NMA) based on the frequency model was adopted to compare the effects of various acupuncture- and moxibustion-related therapies on CRI and to classify these intervention methods according to the results. From the perspective of evidence-based medicine, it is hoped that this study will provide evidence for the clinical selection of the best acupuncture- and moxibustion-related therapy for CRI.

## 2. Methods

### 2.1. Registration

The study protocol was registered (registration number: CRD42022329537) with the International Prospective Register of Systematic Reviews (PROSPERO).

### 2.2. Search strategy

The following databases were searched, starting from inception to June 7, 2022: PubMed, Embase, Web of Science, The Cochrane Library, Wan Fang database, Chinese Biomedical Literature Database (CBM), China National Knowledge Infrastructure (CNKI), and the China Science and Technology Journal Database (VIP). The inclusion criteria and search strategy were established according to the PRISMA protocol guidelines (15). The following three sets of search terms were adopted in English: (neoplasms OR cancer OR tutor OR malignancy) AND (sleep initiation and maintenance disorders OR insomnia OR early awakening OR disorders of initiating and maintaining sleep) AND (acupuncture therapy OR acupuncture OR moxibustion). All searches were limited to human randomized controlled trials (RCTs) and conducted independently by two authors in an electronic database. Relevant Chinese search terms were also searched. To obtain possible related experiments, the references of the original articles and review articles were searched manually. Meanwhile, a combination of subject and free words were used as the search words. The PubMed search strategy is presented in Supplementary Table S1, and appropriate adjustments to this strategy were made for other databases.

### 2.3. Eligibility criteria

1. Adults clinically diagnosed with CRI (>18 years old)
2. Intervention(s): All modalities of acupuncture [AA, EA, SA, transcutaneous electrical acupoint stimulation (TEAS), IN, etc.] and/or moxibustion (direct, indirect, or combined with a needle) or combined with conventional medicine (conventional medicine includes WM and RC. RC refers to comprehensive therapy such as psychotherapy, basic treatment, and emotional care).
3. Comparator(s)/control: sham group (sham acupuncture or sham moxibustion), conventional medicine or other therapies (OT).
4. Primary outcome: Pittsburgh Sleep Quality Index (PSQI) (16).
5. Secondary outcome: Effective rate, adverse events.
6. RCTs only.

### 2.4. Exclusion criteria

1. Studies with repeated data or secondary analysis.
2. Studies from non-RCTs (including animal studies, master and doctoral dissertations, books, conference abstracts, protocols, correspondence, case reports, overviews, and systematic reviews).
3. Non-cancer-related insomnia.
4. The therapy of the intervention group was non-acupuncture or non-moxibustion.
5. The experimental or control group involved traditional Chinese medicine (TCM) or ready-for-use TCM.
6. Outcome indicators do not match.

### 2.5. Study selection and data extraction

Two researchers (LY and SL) performed independent screening of the literature that met the inclusion criteria and conducted cross-checking. Literature screening was carried out according to research type, research objects, intervention/control measures, and outcome indicators. The steps included duplicate checking, primary screening of titles and abstracts, and rescreening of full texts. Two researchers (KW and JL) independently extracted relevant data, including author, title, publication year, journal, country, cancer type, course of the disease, age, sex ratio, randomization method, distribution concealment, blindness, sample size, course of treatment, intervention measures, outcome indicators, and follow-up. After data extraction, two investigators conducted cross-checks. Discussion with a third researcher was done to resolve differences in the literature screening or data extraction process until a consensus was reached. If inadequate or ambiguous data were encountered, we contacted the corresponding author or the first author of the study *via* email to ask for further information.

### 2.6. Risk-of-bias assessment

Two reviewers (DL and YO) referred to the built-in risk bias assessment tool in RevMan 5.3 software (Cochrane Collaboration, Copenhagen, Denmark) to evaluate the risk of bias in the included literature (17). The following seven aspects were assessed: (1) random sequence generation (selection bias), (2) allocation concealment (selection bias), (3) performance bias: blinded implementation (including subjects, investigators, and outcome assessors), (4) detection bias: blinded evaluation of study results, (5) attrition bias: outcome data integrity, (6) reporting bias: selective reporting of results, and (7) other bias. All the above biases were assessed and classified as low, unclear, or high risk. A third researcher was consulted if there was any disagreement in the evaluation process.

### 2.7. Data analysis

After combining direct and indirect evidence from all available RCTs, continuous variables (e.g., PSQI) were reported as mean differences (MDs) with 95% confidence intervals (CIs), while binary categorical variables (e.g., effective rate) were reported as odds ratios (ORs) and 95% CIs. The lower the PSQI score, the better the sleep status, whereas the greater the effective rate, the better the effect. Considering the potential differences within the studies, a random effects model was selected for analysis instead of a fixed effects model (18).

STATA 15.1 (StataCorp, College Station, TX) was used for data analysis and graph drawing. The nodal method was used for the quantification and demonstration of the agreement between direct and indirect comparisons using STATA 15.1. The consistency test was met at *p* > 0.05 (19).

STATA 15.1 was applied to depict network diagrams of different acupuncture- and moxibustion-related therapeutic interventions. As illustrated in the generated network diagrams, each intervention and control condition is represented by a node, and the lines that connect the nodes embody direct head-to-head comparisons between the interventions. The width of the connecting lines and the size of each node are proportional to the number of studies (20).

The intervention hierarchy was summed and reported as a P score, which is regarded as a frequentist analog of the surface under the cumulative ranking curve (SUCRA) values and is used to measure the extent of certainty that one treatment is superior to another, averaged over all competing treatments. The P score ranges from 0 to 1, where 1 indicates that a treatment is the best with the highest degree of certainty and 0 indicates that it is the worst with the lowest degree of certainty. Although the SUCRA or P score can be usefully re-interpreted as the effective percentage of acupuncture interventions, we should still interpret such scores with caution unless there are valid differences between interventions that are clinically meaningful.

### 2.8. Publication bias

In NMA, there are no valid statistical tests other than funnel plots for visual confirmation to detect publication bias. In addition, the traditional funnel plots used for paired meta-analyses are not capable of assessing publication bias in NMA. Consequently, in this review, we attempted to determine the asymmetry of the network funnel plot for the primary outcomes to determine the probability of publication bias.

### 2.9. Quality of evidence

The GRADE approach was adopted to evaluate the confidence of the estimates derived from and NMA of efficacy outcomes (21). In this approach, direct evidence from RCTs starts at high confidence and can be downgraded to levels of moderate, low, and very low confidence based on indirectness, risk of bias, inconsistency (or heterogeneity), imprecision, and/or publication bias. The rating of indirect estimates starts at the lowest rating of the two pairwise estimates that contribute as first-order loops to the indirect estimate but can be downgraded further for intransitivity or imprecision (dissimilarity between studies in terms of clinical or methodological characteristics). Higher ratings, directly or indirectly assessed, apply to the quality of evidence in the NMA and are classified as high, moderate, low, or very low.

## 3. Results

### 3.1. Study identification and selection

A total of 902 studies were retrieved from electronic databases, and no studies were available from other sources. After eliminating duplicates, we searched the titles and abstracts of the remaining 674 studies and re-excluded 248 studies. The remaining 426 studies were read, and 395 were re-excluded (for non-RCTs, unavailable full-text, research object, intervention method, outcome indicators, research type, and repeated data or secondary analysis). Ultimately, 31 studies were included in this meta-analysis. The detailed process is illustrated in Supplementary Figure S1.

### 3.2. Description of study inclusion

We identified 31 RCTs with 3,046 independent participants through a literature search. A PRISMA flowchart is presented in Supplementary Figure S1. Among the 31 studies, the main therapies used in the intervention group were as follows: TEAS, acupuncture and moxibustion (ACU+MOX), AA, IN, RC+IN, AA combined with acupuncture (AA+ACU), SA, RC combined with moxibustion (RC+MOX), and WM combined with acupuncture and moxibustion (WM+ACU+MOX). In addition, the control group mainly involved WM, RC, PSA, AA, ACU, CBT, and RC+AA. Among all studies, only four articles involved acupuncture or moxibustion or direct comparison of different acupuncture therapies (22–25): RC+AA+MOX vs. RC+AA, SA vs. ACU, IN+ OT vs. IN, and RC+AA vs. AA. Moreover, there were 11 cases of acupuncture, moxibustion, or different acupuncture compared with WM, nine cases with RC, and four with PSA, one of them is EA vs. PSA, and EA with a 4-Hz frequency and continuous wave. Diazepam (four trials) was involved in most comparisons, followed by estazolam (three trials), shulediazepam (two trials), and fluoxetine hydrochloride (two trials). The remaining seven RCTs experimented with the efficacy of other interventions: AA, ACU, IN, CBT, RC+AA, and WM+AA. The major acupoints were GV20 (Baihui), SP6 (Sanyinjiao), GV29 (Yintang), PC6 (Neiguan), HT7 (Shenmen), GV24 (Shenting), ST36 (Zusanli), KI1 (Yongquan); moreover, the main auricular points were CO15 (Xin) and TF4 (Shenmen) (Supplementary Table S2). The network plots for the primary outcomes (PSQI) of eligible comparisons are presented in Supplementary Figure S2, whereas the secondary outcomes (effective rate) are presented in Supplementary Figure S3. Although each outcome was included in the systematic review, some interventions were eliminated from the NMA because they were either unrelated or had no available data.

Among the included studies, the fifth edition of the Diagnostic and Statistical Manual of Mental Disorders (DSM-5), the second and third editions of the Chinese Classification of Mental Disorders (CCMD-2 and -3, respectively), the Standard for Diagnosis and Efficacy of Chinese Medicine Syndrome (SDECMS) criteria, and the tenth edition of the International Classification of Diseases (ICD-10) were most often used for diagnosing insomnia. Four studies used the DSM-5 criteria to diagnose insomnia (9, 11, 26, 27); six studies used the CCMD-3 (10, 25, 28–31); one study used the CCMD-2 (23); two studies used the SDECMS criteria (32, 33), and one study used ICD-10 (34). In addition, some studies combined various diagnostic criteria for a more comprehensive diagnosis (35–37).

Of the 2,380 patients whose sex was listed, 1,255 (55.7%) were female. For the included RCTs, the mean sample size was 98.0 (range, 22–220), with the ages of the participants ranging from 31 to 80 years. Three studies (9.67%) were conducted in the United Kingdom (26), Germany (9) and Korea (11), and the remaining 28 (90.33%) were conducted in China (10, 12, 22–25, 27–48)_._ The basic features of the included RCTs are presented in Supplementary Table S2.

### 3.3. Assessment of risk of bias

The assessment of the risk of bias is illustrated in Supplementary Figure S4. Most studies adopted stochastic sequence generation methods with low bias risks (9–12, 22–25, 27, 28, 32, 33, 37, 39–42, 44, 45, 47, 48). Among them, 17 used a random number table (9, 10, 24, 25, 27, 28, 32, 33, 36, 37, 39–42, 44, 45, 48), one used a random sequence (23), two used random sampling (11, 47), and one used computer randomization (22); studies that did not provide a description of the randomization method were given an unclear risk of bias in this domain (30, 35, 38, 43, 46). Five studies were randomized according to the order of visits and were rated as high risk (26, 29, 31, 33, 34). In terms of allocation concealment, two studies used sealed opaque envelopes (11, 26), another study used central randomization (9), and the allocation concealment of the remaining studies was not mentioned. Only Höxtermann et al. (9) conducted blinding of personnel, outcome assessors, and participants. Twenty-nine studies did not provide a description of the blinding strategy of personnel or participants, and some studies were assessed as a high risk given the nature of the intervention (26, 28, 30, 32, 35, 37, 41). The rest were estimated as unclear risks (10–12, 22–24, 27, 29–31, 33, 34, 36, 38, 39, 42–48).

In terms of blinding of outcome assessment, Höxtermann et al. (9)_,_ Lee et al. (11), and Garland et al. (26) described the application of blindness to the assessors of the results and were assessed as low-risk, and the remaining ones were not specified and evaluated as unclear. In terms of outcome data, one study, with a loss rate of 8%, was rated as unclear (32), and another study that did not set the primitive PSQI data preliminarily was rated as high-risk (22). The other 29 studies with complete outcome indicators were rated as low-risk. In the domain of selective reporting, 16 studies were evaluated as having a low risk of bias, of which nine had ethical approval (9, 11, 12, 22, 26–28, 40, 45) and 15 were rated as unclear (23, 24, 30–32, 34–38, 42–44, 46, 47). Regarding other biases, according to our protocol, one study without specific inclusion and exclusion criteria was assessed as high-risk (22). Another study was evaluated as low-risk because it included statistical methods, baseline data, and exclusion criteria (26), and the remaining studies were rated as unclear risk (9–12, 23–25, 27–48). There were no dropouts in 28 studies (9–12, 22, 24–31, 33–46, 48). Dropout cases were mentioned in the remaining studies (23, 32, 47); two studies (23, 47) had a loss rate of less than 5%, and the other was less than 8% (32). However, dropout cases were not considered to have an effect on the study results.

### 3.4. Network meta-analysis

#### 3.4.1. Results of network meta-analysis of Pittsburgh sleep quality index

The network map for the total PSQI score formed two closed loops: RC-AA-RC+AA and WM-SA-ACU (Supplementary Figure S2). All *p*-values for indirect and direct comparisons between all studies were tested for consistency and inconsistency, and the consistency model was acceptable (*p* = 0.8197).

Through the NMA for the total PSQI score, 20 interventions were estimated for the relative effect, including 28 trials. Based on the SUCRA and mean rank (Supplementary Figure S5), the priorities in relation to effectiveness measured by PSQI total score were as follows: TEAS (SUCRA 85.7%), ACU+MOX (79.1%), RC (77.9%), WM (76.5%), PSA (68.1%), AA (62.9%), RC+IN (55.0%), IN (53.3%), SA (50.3%), RC+AA (45.9%), RC+MOX (43.7%), CBT (43.0%), AA+ACU (40.7%), IN+OT (36.8%), EA (35.4%), WM+AA (32.4%), AA+OT (32.2%), ACU (31.1%), RC+AA+MOX (26.6%), WM+ACU+MOX (32.2%).

Based on the comparative efficacy of the treatment (bolding marks supported) presented in Supplementary Table S3, IN (MD −4.08, 95%CI −7.88 to −0.28), SA (MD −3.99, 95%CI −7.94 to −0.04), and WM+AA (MD −4.92, 95%CI −8.75 to −1.09) were all significantly different from WM. Compared with the control group (RC), RC+AA (MD −2.69, 95%CI −4.59 to −0.79), RC+MOX (MD −2.84, 95%CI −5.13 to −0.56), and RC+AA+MOX (MD −4.66, 95%CI −8.95 to −0.36) showed significant differences. Moreover, TEAS was significantly superior to PSA (MD −2.19, 95%CI −4.34 to −0.03) and EA (MD −5.58, 95%CI −10.79 to −0.36).

#### 3.4.2. Results of network meta-analysis of effective rate

The network plot for the NMA which included 16 trials and 12 interventions is shown in Supplementary Figure S3. Based on the SUCRA and mean rank (Supplementary Figure S6), the priorities in relation to effectiveness measured by effective rate were as follows: RC+AA+MOX (SUCRA 95.1%), WM+AA+ACU (94.6%), RC+AA (71.7%), RC+MOX (67.0%), RC+IN (56.3%), AA+ACU (52.6%), WM+AA (48.0%), SA (41.9%), WM+ACU+MOX (39.9%), RC (21.5%), WM (11.0%), ACU+MOX (0.5%).

Based on the comparative efficacy of the treatment presented in Supplementary Table S4, RC+AA (OR 8.11, 95%CI 1.61–40.77), RC+MOX (OR 10.46, 95%CI 1.39–78.88), RC+IN (OR 14.37, 95%CI 1.90–108.76), and WM+AA (OR 20.76, 95%CI 1.63–264.35) showed significant differences compared to RC+AA+MOX. Furthermore, WM+ACU+MOX differed significantly from WM (OR 4.00, 95%CI 1.02–15.68).

#### 3.4.3. Publication bias

Funnel plots for publication bias are shown in Supplementary Figures S7, S8; no significant publication bias was revealed by visually inspecting the funnel plots.

### 3.5. Safety

#### 3.5.1. Adverse events

Seven of the included RCTs assessed AEs (9, 23, 25–27, 37, 45). Two studies reported that the AEs of WM were drowsiness, addiction, drug resistance, and insomnia rebound (27, 37). Five studies involving AA+RC, IN, SA, RC+MOX, and AA+ACU reported that the main AEs were pain, somnolence, tiredness, and small hemorrhages (9, 23, 25–27). One study on TEAS reported serious AEs (45), including seven cases of respiratory depression, eight cases of ventricular tachycardia, and 11 cases of sinus tachycardia. However, this is more likely to be related to preoperative anesthesia than acupuncture. Another study reported that eight patients had cognitive decline during acupuncture treatment (37), which was more likely to be related to the poor mental state of patients after receiving chemotherapy, but not related to acupuncture operations. One study reported no adverse reactions in the RC group (9).

## 4. Quality of evidence

The GRADE levels of the NMA for the total PSQI score were generally medium, low, and very low (Supplementary Table S5). However, the GRADE levels of the NMA for effective rate were generally low to very low (Supplementary Table S6). The main reasons for the degradation were imprecise meta-analysis results and the risk of bias.

## 5. Discussion

### 5.1. Summary of the main results

This is the first study to evaluate the efficacy and safety of different acupuncture- and moxibustion-related treatments for CRI, including 31 RCTs with a large population of patients (*n* = 3,046).

Compared to WM and PSA, acupuncture-related interventions such as TEAS and ACU+MOX were more effective in the PSQI results. In this NMA, according to the therapeutic effect of acupuncture- and moxibustion-related therapies of CRI, the rankings were as follows: TEAS, ACU+MOX, AA, RC+IN, IN, SA, RC+AA, RC+MOX, AA+ACU, IN+OT, EA, WM+AA, AA+OT, ACU, RC+AA+MOX, and WM+ACU+MOX. In terms of comparative effectiveness for the effective rate, we found that RC+AA+MOX had the highest probability of ranking first in treating CRI. Since the secondary outcome (effective rate) involved limited intervention types, it is difficult to make more comprehensive comparisons of the efficacy of different acupuncture- and moxibustion-related treatments. Although few cases of AEs were reported, the incidence of AEs associated with medication treatment, such as drowsiness, addiction, drug resistance, and insomnia rebound, was higher than that of acupuncture treatment. Except for the local pain and hematoma caused by acupuncture- and moxibustion-related treatment, no serious relevant AEs were reported.

### 5.2. Possible explanations for the present findings

Acupuncture and moxibustion are effective and relatively safe treatments for CRI. Among the above measures, AA is one of the recommended treatments. AA is a characteristic acupuncture therapy; in the results of the NMA, the effect was significant, which was consistent with previously reported results (9, 49). Previous studies or reviews focusing on insomnia in breast cancer survivors have evaluated the effects of AA on sleep quality. Furthermore, they have concluded that AA may exert a safe and effective impact in the treatment of breast cancer survivors with insomnia in the short term (22, 50). The sleep-promoting and sleep/wake rhythm-regulating effects of melatonin are attributed to its action on MT1 and MT2 melatonin receptors present in the suprachiasmatic nucleus (SCN) of the hypothalamus (51). Some studies have shown that insomnia is closely related to the decline of central melatonin function (52, 53). The positive feedback loop between parasympathetic vagal nerve excitation and melatonin secretion constitutes the basis for the use of melatonin treatment in insomnia (54, 55). The auricular nail is the only vagus nerve distribution area on the body surface. AA stimulation of the auricular nail area directly stimulates vagal afferent fibers, and the afferent fibers of the auricular branches of the vagus nerve directly project to the nucleus of solitary tract, which then projects directly or indirectly to several nuclei such as the locus coeruleus, parabrachial nucleus, median raphe nuclei, hypothalamus, and other nuclei (56, 57), activating the SCN-pineal gland-melatonin (SCN-PG-MT) axis and promoting the secretion of melatonin to regulate insomnia (58, 59). In addition, the cancer types in this study mainly included breast (9, 11, 12, 22, 24, 26–28, 31, 41, 42, 48), lymph (28, 29, 46), thyroid (11, 34, 40), gastric (31, 37, 41), colorectal (11, 26, 28, 41, 46), and ovarian (27, 28, 31, 41), with breast cancer being the most common type. Whether patients with breast cancer benefit more from acupuncture and whether acupuncture has different curative effects on different cancer types are still unknown.

In addition, acupuncture and moxibustion have remarkable effects in the treatment of CRI. Previous studies have shown that acupuncture and moxibustion can benefit sleep quality and efficiency to a certain extent (27, 32, 60). Several studies have demonstrated effects on various potential neurotransmitters, including melatonin, norepinephrine, endorphin, and gamma-aminobutyric acid (61, 62). Moxibustion is a traditional therapy for insomnia. Previous studies have found that moxibustion has a positive effect on treating insomnia by adjusting the brain’s sleep function, improving sleep quality, and promoting periodicity from light to deep sleep (60, 63). According to TCM theory, the key to the treatment of insomnia by acupuncture and moxibustion is to regulate the excess and deficiency of yin and yang according to the attributes of syndromes to balance the yin and yang of the body and restore its normal physiological function. However, there are few reports on the underlying mechanisms of acupuncture and moxibustion of CRI. Therefore, the mechanism of acupuncture and moxibustion combination therapy for treating CRI requires further exploration and confirmation.

Surprisingly, TEAS has a positive effect in treating CRI and is superior to PSA. TEAS combines traditional acupuncture therapy with modern transcutaneous electrical stimulation technology, which is an important supplement and alternative medical method to acupuncture therapy (64). The effect of TEAS is similar to that of EA, with similar peripheral and central mechanisms. Through the sensory transmission of the meridians, qi and blood can be dredged, and the yin and yang of the zang-fu viscera can be adjusted (65). In previous reports, Dong et al. (66) and Ding et al. (67) demonstrated that TEAS could effectively improve sleep quality in insomnia patients. However, the choice of stimulation acupoints, treatment frequency, current intensity, and duration may cause certain heterogeneity (68).

RC is a comprehensive therapy, including psychotherapy, basic treatment, and emotional care, which play an essential role in treating CRI. For example, emotional care in TCM can help patients with insomnia relieve tension, anxiety, depression, and other negative emotions to help them sleep in a relaxed and natural state (69). Low-resistance thought induction psychology in modern TCM psychotherapy allows patients to enter a specific state from waking to sleeping through the induction of language and behavior, which is increasingly widely used in insomnia treatment (70, 71). In our results, the ranking of RC was relatively high, whether alone or in combination with drugs or acupuncture- and moxibustion-related therapies, showing a positive effect.

Based on the results of our review, we found that the most used acupoints for treating CRI are GV20, SP6, and HT7. GV20-SP6-HT7 is also the most used combination of main acupoints for treating insomnia (72). Studies have shown that acupuncture at GV20 can significantly improve the expression levels of clock genes and amino acid neurotransmitters in the brain tissue of rats with insomnia, thus improving sleep (73). Acupuncture at SP6 stimulates cognitive-emotional brain areas such as the anterior cingulate cortex and thalamus after sleep deprivation and promotes sleep (74). In addition, acupuncture at SP6-HT7 can downregulate serum adrenocorticotropic hormone (ACTH) and cortisol levels in patients with insomnia, inhibit the hyperactive hypothalamic–pituitary–adrenal axis, increase serum melatonin levels, and improve the function of the SCN-PG-MT system, thus regulating sleep (58, 75). Therefore, acupuncture at GV20, SP6, and HT7 may exert a calming effect and adjust the sleep state by regulating the activity of the sleep–wake center and related factors.

Acupuncture and moxibustion are two of the most commonly used non-pharmaceutical traditional Chinese medicine therapies in clinical practice. Acupuncture uses different needles or instruments to stimulate acupoints for therapeutic purposes, mainly by mechanical stimulation. Moxibustion uses artemisia argyi or other medicines to stimulate the acupoints by cauterizing them to regulate the body’s functions, mainly including thermal effects, light radiation effects, and pharmacological actions of artemisia argyi (76). Based on the characteristics of the moxibustion spectrum, the selection of different wavelength light stimulation sources to simulate the light radiation effect of moxibustion stimulation has also gradually attracted attention. It was found that 10.6 μm wavelength laser acupuncture is close to the peak of the infrared radiation spectrum of moxibustion and human acupuncture points. Its efficacy is similar to traditional moxibustion, producing a moxibustion-like thermal effect without smoke and smell (77–79).

### 5.3. Limitations of included studies

#### 5.3.1. Evidence and methodological quality

The GRADE profile for the PSQI and the effective rate showed that the evidence quality of all results was mostly low, which was mainly due to methodological limitations such as randomness, risk of bias, blindness, and allocation concealment in reporting results. Therefore, the overall quality was low. Among the 31 enrolled RCTs, five did not provide a detailed description of the randomization process (30, 35, 38, 43, 46). In addition, only three RCTs listed information on allocation concealment (9, 11, 26). Only one RCT met the blinding requirement of treatment allocation (9) and two RCTs met the blinding requirement of outcome assessment (11, 26). Additionally, no description was available to identify the existence of selective reporting bias in the included studies. These various types of bias may have contributed to the false-positive results. Moreover, only one RCT fully mentioned statistical methods, baseline data, and exclusion criteria. In addition, the heterogeneity in diagnostic criteria should be considered when interpreting the results between studies. Therefore, the statistical analysis power of the included RCTs might be extremely low.

#### 5.3.2. Inconsistent interventions

Compared with the interventions involved in this review, the number of the included RCTs is small, which leads to limitations of most outcomes, especially in some interventions involving only one or two RCTs. Moreover, the included RCTs also varied in terms of the number of sessions, frequency, selection of acupoints, number of acupoints, duration of acupuncture, time for needle retention, and needling depth, all of which might have contributed to bias. CBT is the first-line treatment for insomnia. However, due to the lack of inclusion in this review and the incomplete coverage of acupuncture or moxibustion, we cannot comprehensively compare CBT with other acupuncture or moxibustion treatments, and the final ranking results should be treated with caution.

#### 5.3.3. Limited outcomes

Due to the limited outcomes included in the study, it was impossible to comprehensively evaluate the difference in the therapeutic effect of acupuncture on CRI. Moreover, only seven studies reported AEs (9, 23, 25–27, 37, 45), and only six studies mentioned follow-up (11, 25–27, 30, 32). Thus, the safety and long-term effects of acupuncture- and moxibustion-related therapies for CRI require further exploration.

#### 5.3.4. Lack of health economic data

The included studies have no health economic data or related health economic analysis reports.

### 5.4. Strengths and limitations of this review

Network meta-analysis is a precious method that enables the selection of the most efficient ones among multiple treatment options. Complementary and alternative therapies are important and effective for individuals with CRI. To the best of our knowledge, few studies have attempted to estimate the comparative effectiveness of various acupuncture- and moxibustion-related treatments. Based on the current evidence, the advantage of this review lies in applying the NMA method to compare the effectiveness and safety of different acupuncture treatments for CRI. The results are of great benefit to patients, clinicians, and policymakers in making decisions regarding ideal acupuncture- and moxibustion-related therapies for treating CRI.

There are also limitations of our study. First, the search languages of literature were limited to English and Chinese articles, excluding studies published in other languages. Biased outcomes may have been attributed to this language limitation. Second, due to the unclear follow-up description in the included literature, it was impossible to further explore the long-term effects of acupuncture on CRI. Third, the quality of the evidence was not ideal because of the imperfect study design and the limited number of included trials.

Analyzing the included studies, we found that many studies lacked attention to the course and follow-up of CRI. We hope that there will be more high-quality RCTs involving more acupuncture and moxibustion treatments to further explore the effects of acupuncture and moxibustion in treating CRI, including safety, effectiveness, stability, and durability.

## 6. Conclusion

Acupuncture and moxibustion are effective and relatively safe treatments for CRI. The relatively conservative recommended order of acupuncture and moxibustion-related therapies for CRI is as follows: TEAS, acupuncture and moxibustion, and AA. Among these, TEAS had the highest probability of ranking first in treating CRI. Nevertheless, the methodological quality of the included studies was generally poor, and further well-designed, large-scale, high-quality RCTs are required to verify our findings.

## Data availability statement

The original contributions presented in the study are included in the article/Supplementary material, further inquiries can be directed to the corresponding author.

## Author contributions

YO, DL, and LZ conceived and designed the study. YO, DL, and XN searched the databases. SL and LY participated in the study selection. SL, LY, KW, and JL extracted the data. YO, DL, XN, CF, YY, XW, LW, ZT, and JR interpreted and assessed the data. YO and DL depicted tables and figures. YO, DL, XN, and JR drafted the manuscript. LZ revised the manuscript. All authors contributed to the article and approved the submitted version.

## Funding

This work was funded by the Project of the Science and Technology Department of Sichuan Province (Grant numbers 2021ZYD0103 and 2020YFS0304).

## Conflict of interest

The authors declare that the research was conducted in the absence of any commercial or financial relationships that could be construed as a potential conflict of interest.

## Publisher’s note

All claims expressed in this article are solely those of the authors and do not necessarily represent those of their affiliated organizations, or those of the publisher, the editors and the reviewers. Any product that may be evaluated in this article, or claim that may be made by its manufacturer, is not guaranteed or endorsed by the publisher.

## Abbreviations

CRI: cancer-related insomnia
RCTs: randomized controlled trials
PSQI: Pittsburgh Sleeps Quality Index
AEs: adverse events
CBM: Chinese Biomedical Literature Database
CNKI: China National Knowledge Infrastructure
VIP: the China Science and Technology Journal Database
MD: mean difference
OR: odds ratio
CI: confidence intervals
NMA: network meta-analysis
SUCRA: surface under the cumulative ranking curve
TCM: traditional Chinese medicine
WM: western medicine
RC: routine care
AA: auricular acupuncture
CBT: cognitive behavioral therapy
ACU+MOX: acupuncture and moxibustion
AA+ACU: auricular acupuncture combined with acupuncture
RC+AA: routine care combined with auricular acupuncture
RC+MOX: routine care combined with moxibustion
RC+IN: routine care combined with intradermal needling
RC+AA+MOX: routine care combined with auricular acupuncture and moxibustion
WM+AA+MOX: western medicine combined with auricular acupuncture and moxibustion
WM+ACU+MOX: western medicine combined with acupuncture and moxibustion
WM+AA: western medicine combined with auricular acupuncture
SA: scalp-acupuncture
ACU: acupuncture
EA: electro-acupuncture
IN: intradermal needling
IN+OT: intradermal needling combined with other therapies
TEAS: transcutaneous electrical acupoint stimulation
AA+OT: acupuncture combined with other therapies
PSA: placebo-sham acupuncture
