# Supplementary material for: Acupuncture and moxibustion in patients with cancer-related insomnia: A systematic review and network meta-analysis
Source: Front Psychiatry. 2023 Feb 16;14:1108686. doi: 10.3389/fpsyt.2023.1108686 (PMC9979218; doi:10.3389/fpsyt.2023.1108686)
Supplement: Supplementary file 1 [file Data_Sheet_1.docx]

**Supplementary Figures**

[FIGURE 1| PRISMA flow chart 2](#_Toc119070643)

[FIGURE 2| Network map for PSQI total score 3](#_Toc119070644)

[FIGURE 3| Network map for effective rate total score 4](#_Toc119070645)

[FIGURE 4| Bias risk assessment map 5](#_Toc119070646)

[FIGURE 5| SUCRA for PSQI total score 5](#_Toc119070647)

[FIGURE 6| SUCRA for effective rate total score 6](#_Toc119070648)

[FIGURE 7| Funnel plot on publication bias of PSQI 7](#_Toc119070649)

[FIGURE 8| Funnel plot on publication bias of effective rate 8](#_Toc119070650)

FIGURE 1| PRISMA flow chart


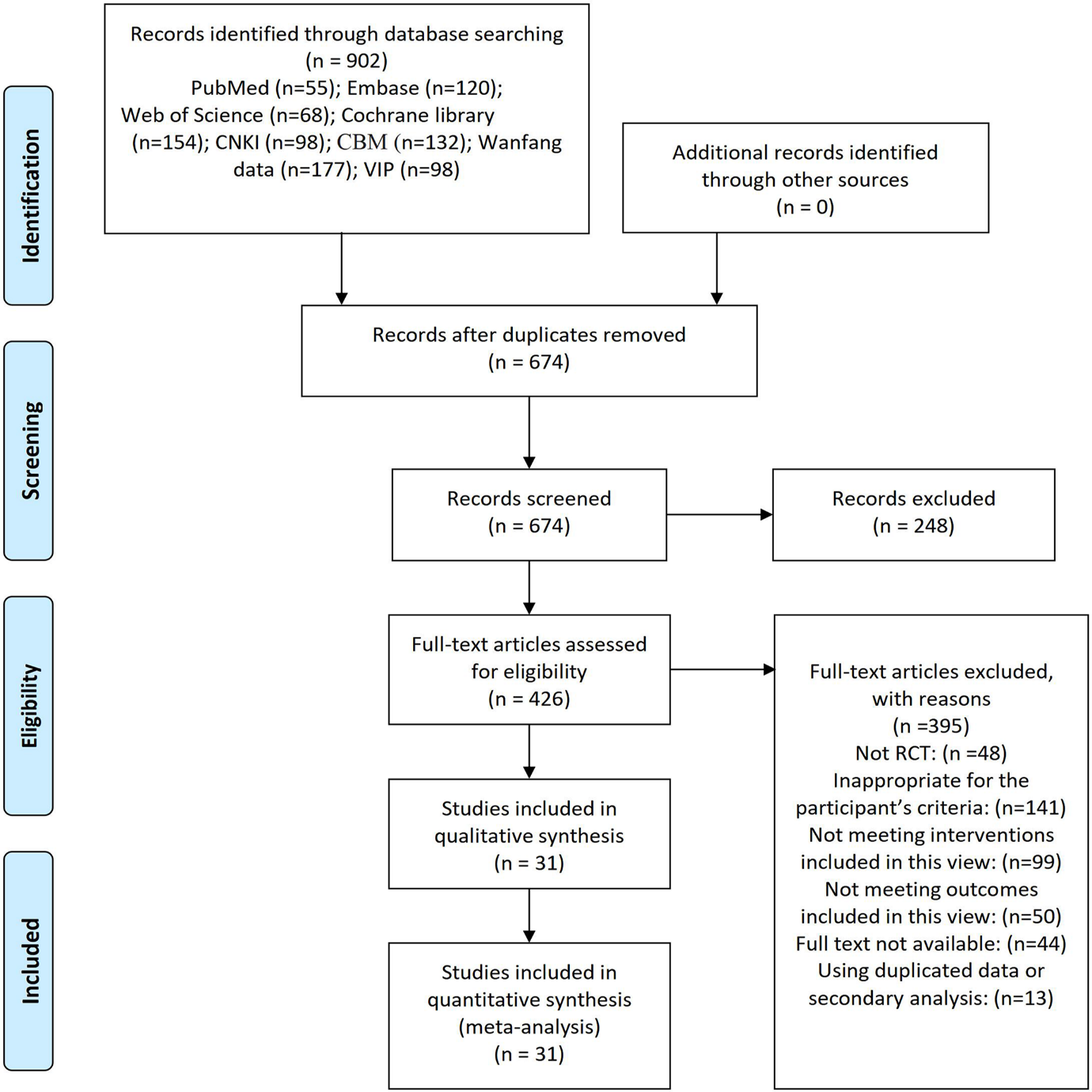


CNKI, China national knowledge infrastructure; VIP, Technology Journal database; CBM, Chinese Biomedical Literature Database; RCT, randomized controlled trial;

**FIGURE 2|** Network map for PSQI total score

**
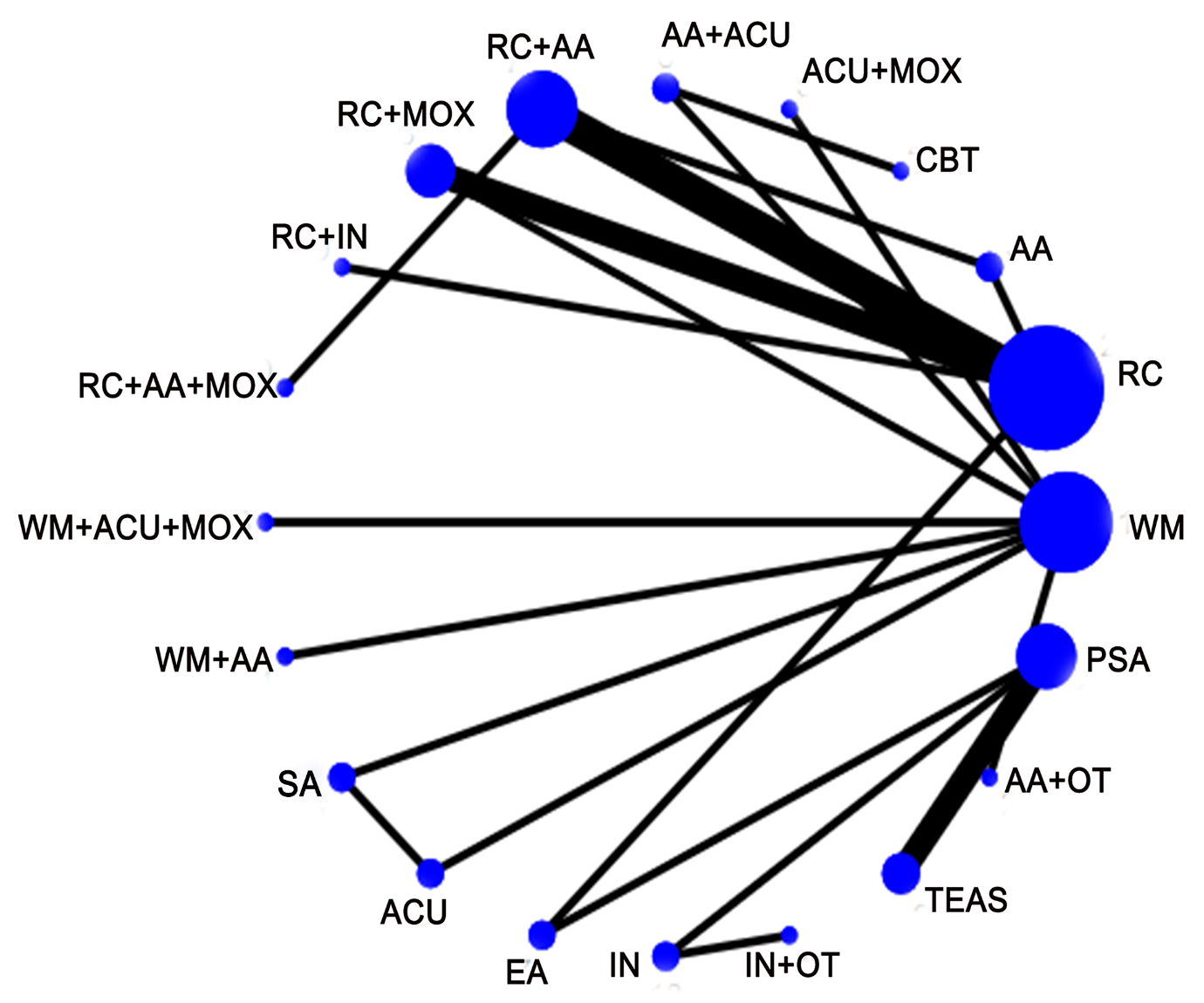
**

WM, western medicine; RC, routine care; AA, auricular acupuncture; CBT, cognitive behavioral therapy; ACU+MOX, acupuncture and moxibustion; AA+ACU: auricular acupuncture combined with acupuncture; RC+AA: routine care combined with auricular acupuncture; RC+MOX: routine care combined with moxibustion; RC+IN: routine care combined with intradermal needling; RC+AA+MOX: routine care combined with auricular acupuncture and moxibustion; WM+ACU+MOX: western medicine combined with acupuncture and moxibustion; WM+AA: western medicine combined with auricular acupuncture; SA: scalp-acupuncture; ACU: acupuncture; EA: electro-acupuncture; IN: intradermal needling; IN+OT: intradermal needling combined with other therapies; TEAS: transcutaneous electrical acupoint stimulation; AA+OT: acupuncture combined with other therapies; PSA: placebo-sham acupuncture.

**FIGURE 3|** Network map for effective rate total score


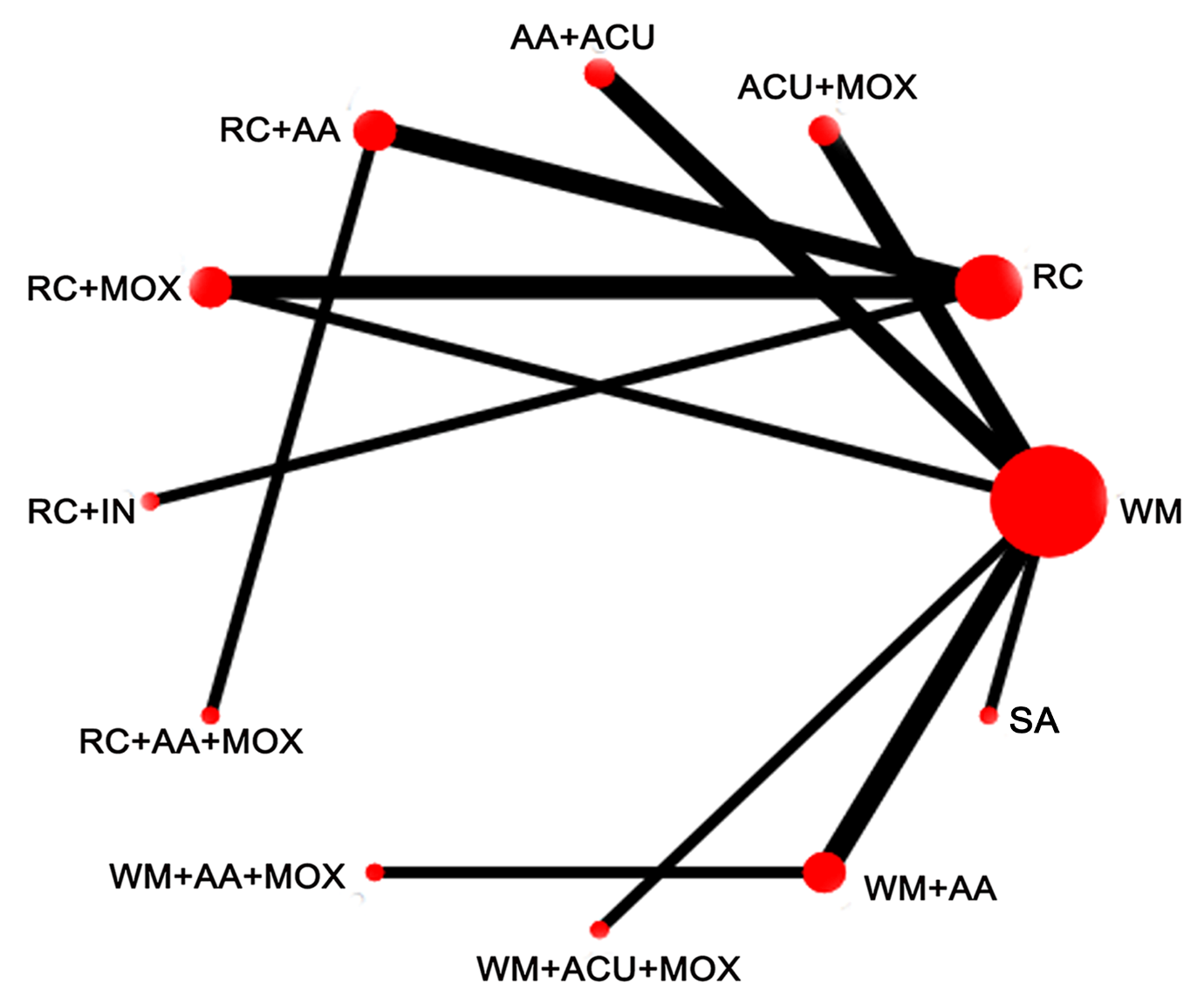


WM, western medicine; RC, routine care; ACU+MOX, acupuncture and moxibustion; AA+ACU: auricular acupuncture combined with acupuncture; RC+AA: routine care combined with auricular acupuncture; RC+MOX: routine care combined with moxibustion; RC+IN: routine care combined with intradermal needling; RC+AA+MOX: routine care combined with auricular acupuncture and moxibustion; WM+AA+MOX: western medicine combined with auricular acupuncture and moxibustion; WM+ACU+MOX: western medicine combined with acupuncture and moxibustion; WM+AA: western medicine combined with auricular acupuncture; SA: scalp-acupuncture.

FIGURE **4|Bias risk assessment map**


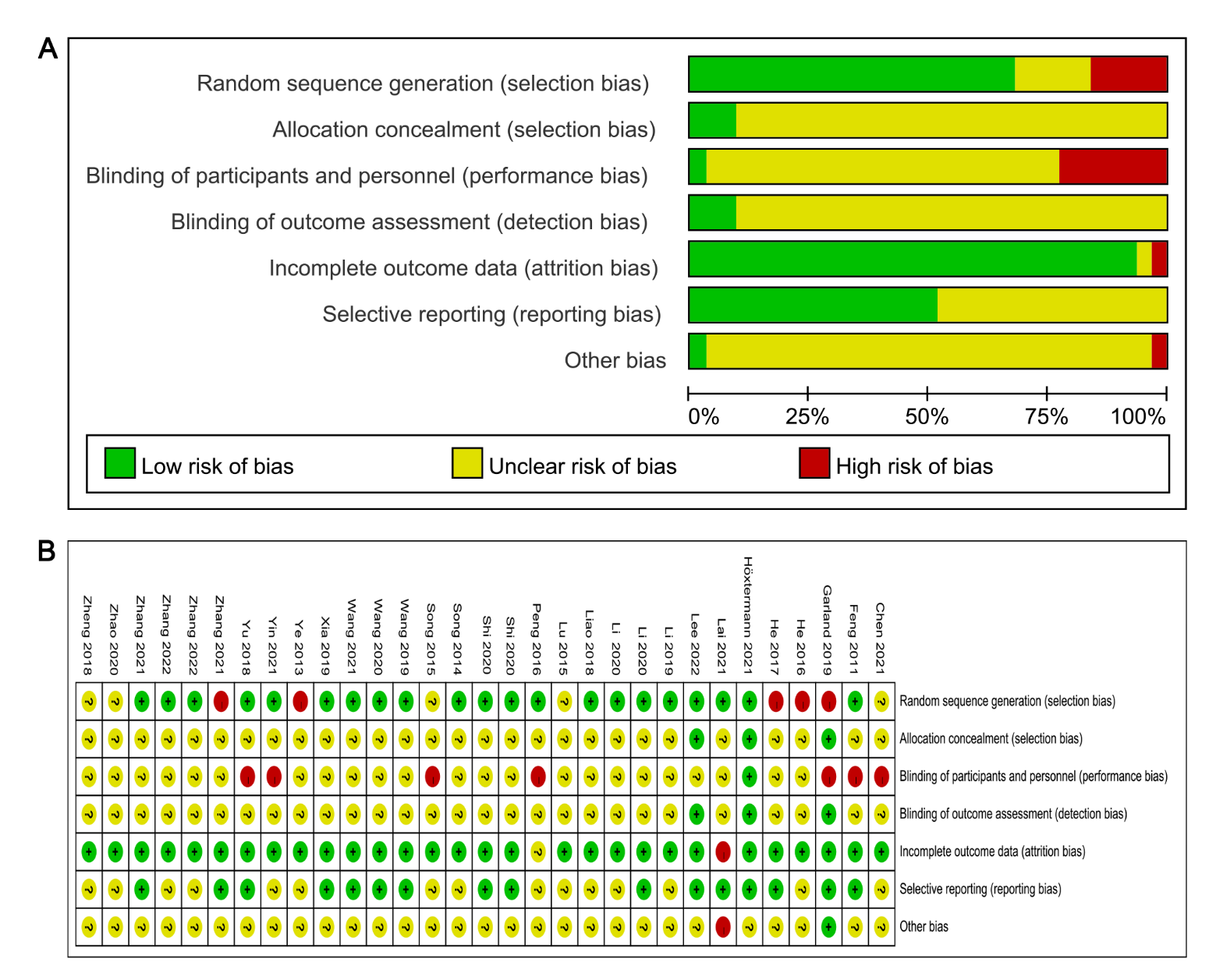


1. Risk of bias graph. ****(B)**** Risk of bias summary.

**FIGURE 5|** SUCRA for PSQI total score


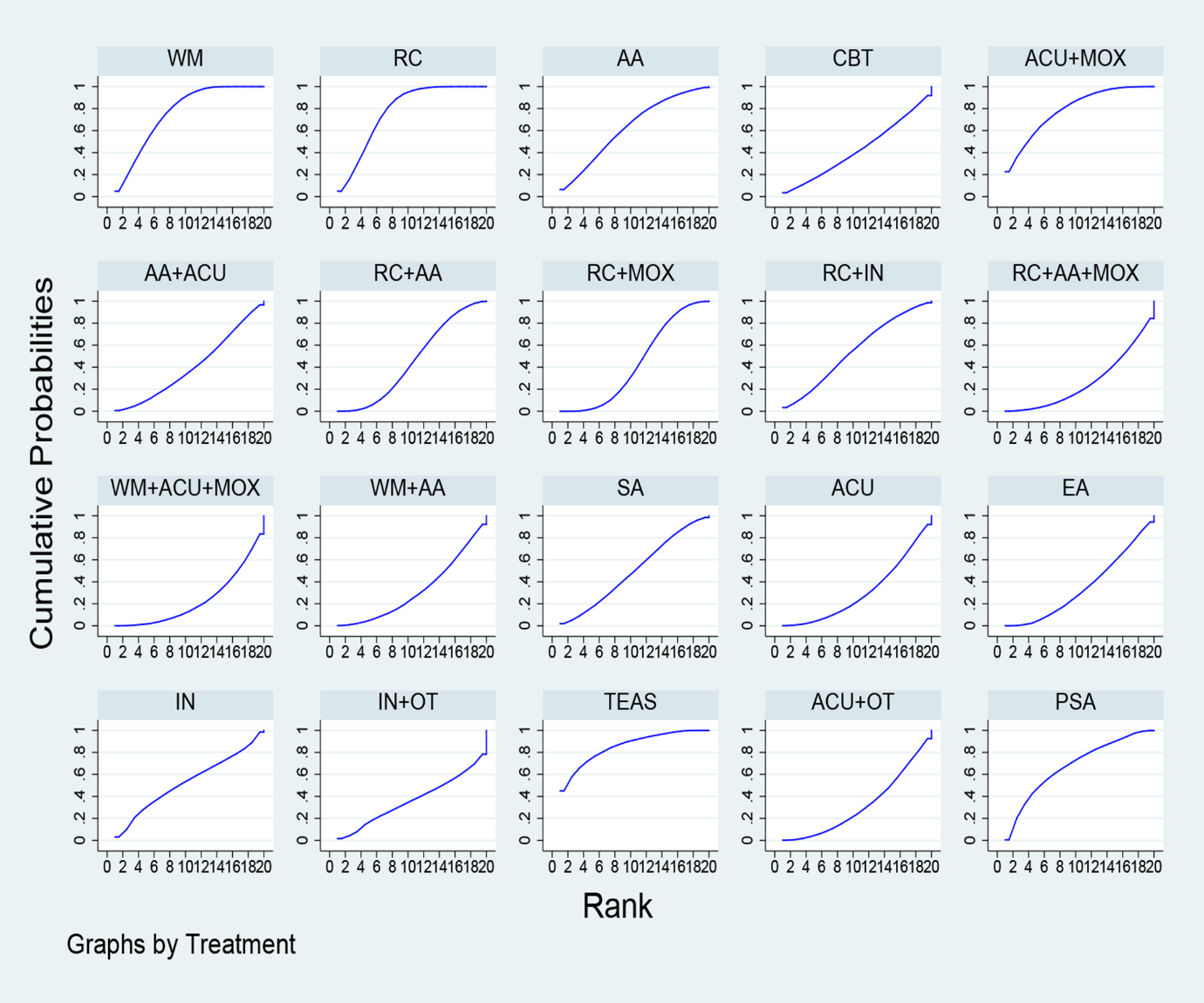


WM, western medicine; RC, routine care; AA, auricular acupuncture; CBT, cognitive behavioral therapy; ACU+MOX, acupuncture and moxibustion; AA+ACU: auricular acupuncture combined with acupuncture; RC+AA: routine care combined with auricular acupuncture; RC+MOX: routine care combined with moxibustion; RC+IN: routine care combined with intradermal needling; RC+AA+MOX: routine care combined with auricular acupuncture and moxibustion; WM+ACU+MOX: western medicine combined with acupuncture and moxibustion; WM+AA: western medicine combined with auricular acupuncture; SA: scalp-acupuncture; ACU: acupuncture; EA: electro-acupuncture; IN: intradermal needling; IN+OT: intradermal needling combined with other therapies; TEAS: transcutaneous electrical acupoint stimulation; AA+OT: acupuncture combined with other therapies; PSA: placebo-sham acupuncture.

**FIGURE 6|** SUCRA for effective rate total score


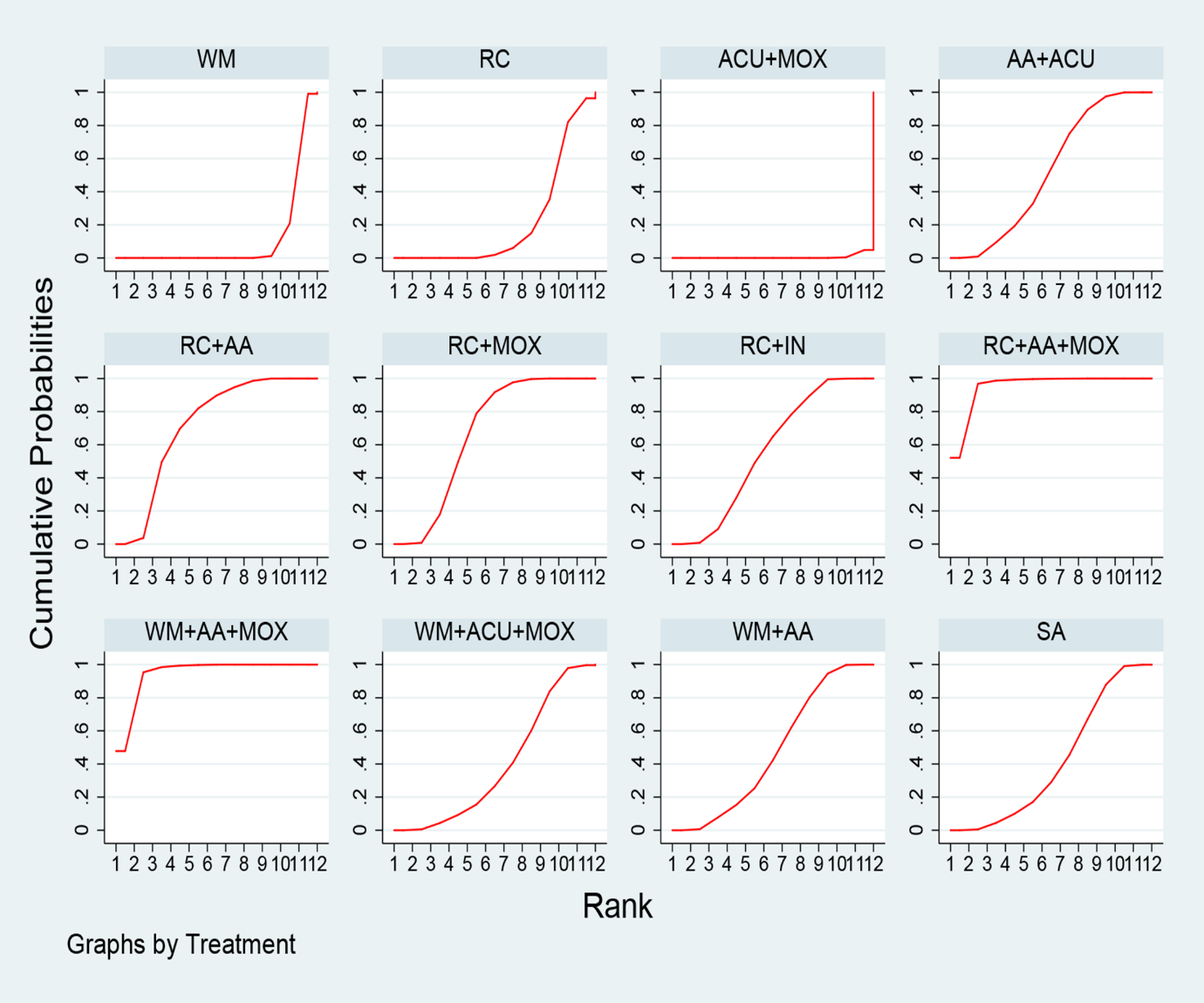
WM, western medicine; RC, routine care; ACU+MOX, acupuncture and moxibustion; AA+ACU: auricular acupuncture combined with acupuncture; RC+AA: routine care combined with auricular acupuncture; RC+MOX: routine care combined with moxibustion; RC+IN: routine care combined with intradermal needling; RC+AA+MOX: routine care combined with auricular acupuncture and moxibustion; WM+AA+MOX: western medicine combined with auricular acupuncture and moxibustion; WM+ACU+MOX: western medicine combined with acupuncture and moxibustion; WM+AA: western medicine combined with auricular acupuncture; SA: scalp-acupuncture.

**FIGURE 7|** Funnel plot on publication bias of PSQI


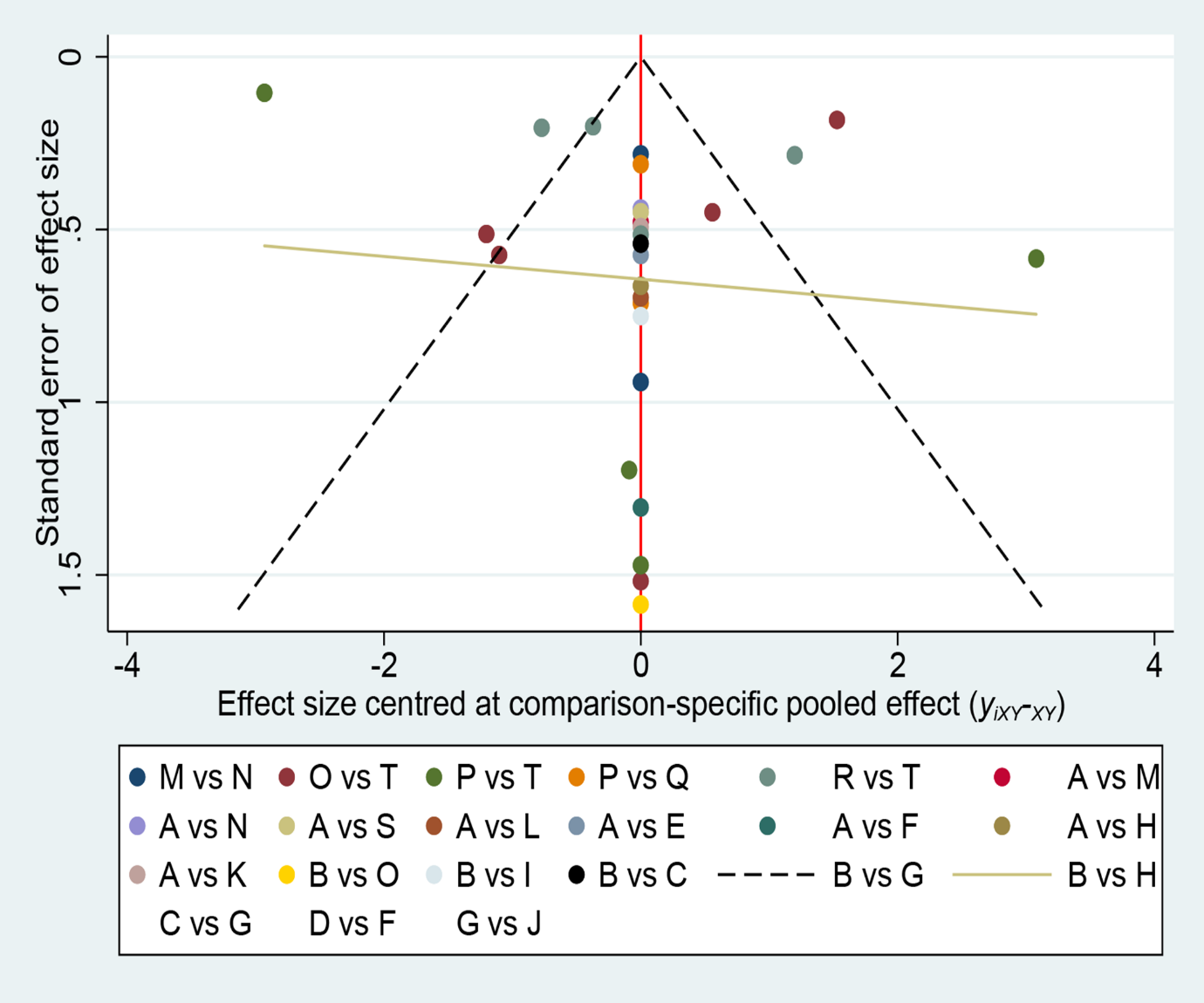


A:WM, western medicine; B:RC, routine care; C:AA, auricular acupuncture; D:CBT, cognitive behavioral therapy; E:ACU+MOX, acupuncture and moxibustion; F:AA+ACU: auricular acupuncture combined with acupuncture; G:RC+AA: routine care combined with auricular acupuncture; H:RC+MOX: routine care combined with moxibustion; I:RC+IN: routine care combined with intradermal needling; J:RC+AA+MOX: routine care combined with auricular acupuncture and moxibustion; K:WM+ACU+MOX: western medicine combined with acupuncture and moxibustion; L:WM+AA: western medicine combined with auricular acupuncture; M:SA: scalp-acupuncture; N:ACU: acupuncture; O:EA: electro-acupuncture; P:IN: intradermal needling; Q:IN+OT: intradermal needling combined with other therapies; R:TEAS: transcutaneous electrical acupoint stimulation; S:AA+OT: acupuncture combined with other therapies; T:PSA: placebo-sham acupuncture.

**FIGURE 8|** Funnel plot on publication bias of effective rate


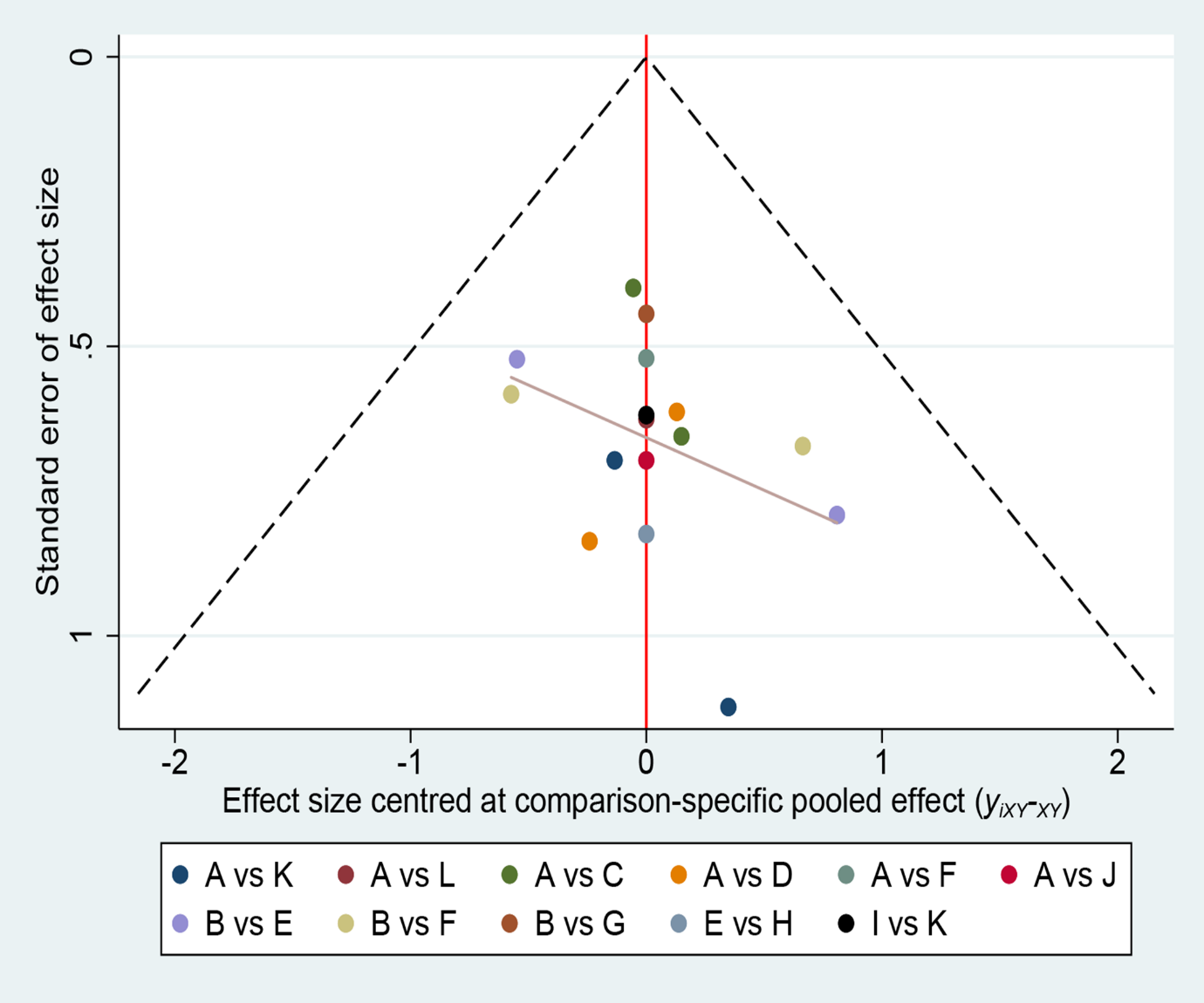


A:WM, western medicine; B:RC, routine care; C:ACU+MOX, acupuncture and moxibustion; D:AA+ACU: auricular acupuncture combined with acupuncture; E:RC+AA: routine care combined with auricular acupuncture; F:RC+MOX: routine care combined with moxibustion; G:RC+IN: routine care combined with intradermal needling; H:RC+AA+MOX: routine care combined with auricular acupuncture and moxibustion; I:WM+AA+MOX: western medicine combined with auricular acupuncture and moxibustion; J:WM+ACU+MOX: western medicine combined with acupuncture and moxibustion; K:WM+AA: western medicine combined with auricular acupuncture; L:SA: scalp-acupuncture.
