# Supplementary material for: Acupuncture and moxibustion in patients with cancer-related insomnia: A systematic review and network meta-analysis
Source: Front Psychiatry. 2023 Feb 16;14:1108686. doi: 10.3389/fpsyt.2023.1108686 (PMC9979218; doi:10.3389/fpsyt.2023.1108686)
Supplement: Supplementary file 2 [file Data_Sheet_2.docx]

**Supplementary Tables**

[TABLE 1| Search Strategy for the PubMed Database 2](#_Toc119062184)

[TABLE 2| Basic Features of Included RCTs 4](#_Toc119062185)

[TABLE 3| Head-to-head comparisons for PSQI of the different interventions 11](#_Toc119062186)

[TABLE 4| Head-to-head comparisons for an effective rate of different interventions 13](#_Toc119062187)

[TABLE 5| Evidence Quality of PSQI Score 14](#_Toc119062188)

[TABLE 6| Evidence Quality of  effective rate 20](#_Toc119062189)

TABLE 1| Search Strategy for the PubMed Database

| **#1** | **“Acupuncture Therapy”[MeSH Terms]** |
| --- | --- |
| **#2** | **(((((((((Acupuncture Treatment[Title/Abstract]) OR (Acupuncture Treatments[Title/Abstract])) OR (Treatment, Acupuncture[Title/Abstract])) OR (Therapy, Acupuncture[Title/Abstract])) OR (Pharmacoacupuncture Treatment[Title/Abstract])) OR (Treatment, Pharmacoacupuncture[Title/Abstract])) OR (Pharmacoacupuncture Therapy[Title/Abstract])) OR (Therapy, Pharmacoacupuncture[Title/Abstract])) OR (Acupotomy[Title/Abstract])) OR (Acupotomies[Title/Abstract])** |
| **#3** | **(acupunctur*[Title/Abstract] OR electroacupunctur*[Title/Abstract] OR electro-acupunctur*[Title/Abstract] OR acupoint*[Title/Abstract] OR electric acupunctur*[Title/Abstract] OR acu-point*[Title/Abstract] OR acupress*[Title/Abstract])** |
| **#4** | **(meridian*[Title/Abstract]) OR (non-meridian[Title/Abstract] OR trigger[Title/Abstract])** |
| **#5** | **(((((((((((((zhenjiu[Title/Abstract]) OR (zhen jiu[Title/Abstract])) OR (zhenci[Title/Abstract])) OR (zhen ci[Title/Abstract])) OR (cizhen[Title/Abstract])) OR (dianzhen[Title/Abstract])) OR (dian zhen[Title/Abstract])) OR (zhen ya[Title/Abstract])) OR (er zhen[Title/Abstract])) OR (ti zhen[Title/Abstract])) OR (she zhen[Title/Abstract])) OR (tou pi zhen[Title/Abstract])) OR (zue wei[Title/Abstract])) OR (pi nei zhen[Title/Abstract])** |
| **#6** | **((((((((((((((moxibustion[Title/Abstract]) OR (fire needle[Title/Abstract])) OR (fire acupuncture[Title/Abstract])) OR (warm acupuncture[Title/Abstract])) OR (warm needle[Title/Abstract])) OR (needle warming moxibustion[Title/Abstract])) OR (heat sensitive moxibustion[Title/Abstract])) OR (ear needle[Title/Abstract])) OR (auricular needle[Title/Abstract])) OR (wrist ankle needle[Title/Abstract])) OR (acupoint embedding[Title/Abstract])) OR (acupoint injection[Title/Abstract])) OR (acupoint sticking[Title/Abstract])) OR (intradermal needling[Title/Abstract])) OR (scalp acupuncture[Title/Abstract])** |
| **#7** | **(((((((((((((transcutaneous electric$ nerve stimulation[Title/Abstract]) OR (percutaneous electric$ stimulation[Title/Abstract])) OR (transcutaneous electric$ stimulation[Title/Abstract])) OR (transdermal electrostimulation[Title/Abstract])) OR (transcutaneous electric$ acupoint stimulation[Title/Abstract])) OR (neuromuscular electric$ stimulation[Title/Abstract])) OR (functional electric$ stimulation[Title/Abstract])) OR (transcranial direct current stimulation[Title/Abstract])) OR (TENS[Title/Abstract])) OR (PENS[Title/Abstract])) OR (TEAS[Title/Abstract])) OR (NMES[Title/Abstract])) OR (FES[Title/Abstract])) OR (tDCS[Title/Abstract])** |
| **#8** | **#1 OR #2 OR #3 OR #4 OR #5 OR #6 OR #7** |
| **#9** | **“Neoplasms”[MeSH Terms]** |
| **#10** | **((((((((((((((((((Tumor[Title/Abstract]) OR (Neoplasm[Title/Abstract])) OR (Tumors[Title/Abstract])) OR (Neoplasia[Title/Abstract])) OR (Neoplasias[Title/Abstract])) OR (Cancer[Title/Abstract])) OR (Cancers[Title/Abstract])) OR (Malignant Neoplasm[Title/Abstract])) OR (Malignancy[Title/Abstract])) OR (Malignancies[Title/Abstract])) OR (Malignant Neoplasms[Title/Abstract])) OR (Neoplasm, Malignant[Title/Abstract])) OR (Neoplasms, Malignant[Title/Abstract])) OR (Benign Neoplasms[Title/Abstract])) OR (Benign Neoplasm[Title/Abstract])) OR (Neoplasms, Benign[Title/Abstract])) OR (Neoplasm, Benign[Title/Abstract])) OR (zhongliu[Title/Abstract])) OR (aizheng[Title/Abstract])** |
| **#11** | **#9 or #10** |
| **#12** | **“Sleep Initiation and Maintenance Disorders”[MeSH Terms]** |
| **#13** | **((((((((((((((((((((((((((Disorders of Initiating and Maintaining Sleep[Title/Abstract]) OR (DIMS (Disorders of Initiating and Maintaining Sleep[Title/Abstract]))) OR (Early Awakening[Title/Abstract])) OR (Awakening, Early[Title/Abstract])) OR (Nonorganic Insomnia[Title/Abstract])) OR (Insomnia, Nonorganic[Title/Abstract])) OR (Primary Insomnia[Title/Abstract])) OR (Insomnia, Primary[Title/Abstract])) OR (Transient Insomnia[Title/Abstract])) OR (Insomnia, Transient[Title/Abstract])) OR (Rebound Insomnia[Title/Abstract])) OR (Insomnia, Rebound[Title/Abstract])) OR (Secondary Insomnia[Title/Abstract])) OR (Insomnia, Secondary[Title/Abstract])) OR (Sleep Initiation Dysfunction[Title/Abstract])) OR (Dysfunction, Sleep Initiation[Title/Abstract])) OR (Dysfunctions, Sleep Initiation[Title/Abstract])) OR (Sleep Initiation Dysfunctions[Title/Abstract])) OR (Sleeplessness[Title/Abstract])) OR (Insomnia Disorder[Title/Abstract])) OR (Insomnia Disorders[Title/Abstract])) OR (Insomnia[Title/Abstract])) OR (Insomnias[Title/Abstract])) OR (Chronic Insomnia[Title/Abstract])) OR (Insomnia, Chronic[Title/Abstract])) OR (Psychophysiological Insomnia[Title/Abstract])) OR (Insomnia, Psychophysiological[Title/Abstract])** |
| **#14** | **#12 or #13** |
| **#15** | **((((((((((((((groups[Title/Abstract]) OR (trial[Title/Abstract])) OR (randomized controlled trial[Title/Abstract])) OR (controlled clinical trial[Title/Abstract])) OR (random allocation[Title/Abstract])) OR (randomized[Title/Abstract])) OR (randomly[Title/Abstract])) OR (double-blind method[Title/Abstract])) OR (single-blind method[Title/Abstract])) OR (clinical trial[Title/Abstract])) OR ((double$ adj blind$[Title/Abstract])) OR (placebo[Title/Abstract])) OR (randomized[Title/Abstract])) OR (Randomized Controlled Trial[Publication Type])) OR (controlled clinical trial[Publication Type])** |
| **#16** | **#8 and #11 and #14 and #15** |

TABLE 2| Basic Features of Included RCTs

| **Study** | **Age (E/C)** | **Male (E/C)** | **Female (E/C)** | **Interventions (E/C)** | | **Cancer type（s）** | **Diagnostic criteria** | **Period of treatment (E/C)** | **Outcomes** | **Acupuncture points** |
| --- | --- | --- | --- | --- | --- | --- | --- | --- | --- | --- |
|  |  |  |  | **E** | **C** |  |  |  |  |  |
| **Chen (35)**  **2021** | 62.85±5.61  /62.56±5.57Y | 18/14 | 17/15 | AA  +ACU | WM | LCA | Guidelines for Clinical Research of New Chinese Medicines and CCMD-2 | 2 times a week (4W)  /Once daily (8W) | 1.effective rate; 2.PSQI | The following are auricular points:  TF4 *Shenmen* 神门,  CO15 *Xin* 心,  AT4 *Pizhixia* 皮质下,  CO18 *Neifenmi* 内分泌. |
| **FENG et al. (28)**  **2011** | 63.8±5.47  /63.6±4.26Y | 26/14 | 27/13 | ACU | WM | LCA, GC, BC, CRC, LC, CCA, OC | CCMD-3 | Once daily (4W)  /NR | 1.PSQI; | ST40 *Fenglong* 丰隆,  SP9 *Yinlingquan* 阴陵泉,  SP10 *Xuehai* 血海,  SP6 *Sanyinjiao* 三阴交,  GV29 *Yintang* 印堂,  GV20 *Baihui* 百会,  EX-HN1 *Sishencong* 四神聪,  PC6 *Neiguan* 内关,  HT7 *Shenmen* 神门. |
| **He et al. (29)**  **2017** | 46.00±13.67  /45.81±13.82Y | 21/11 | 18/14 | RC  +MOX | RC | LC | CCMD-3 | Once daily (NR)  /NR | 1.PSQI; | HT7 *Shenmen* 神门. |
| **Höxtermann et al.(9)**  **2021** | 56.58±7.9  /54.8±8.3Y | NR | NR | AA | RC | BC | DSM-5 | Two times a week (5W)  /NR | 1.PSQI; 2.AE | The following are auricular points:  postantitragal belt,  helix channel,  TF4 *Shenmen* 神门. |
| **Lai et al. (22)**  **2021** | 55.5±5.7  /54.5±5.5Y | N | 77 | RC+AA | AA | BC | NR | Press 3-5 times a day, replacement once in two days (NR)  /Press 3-5 times a day, replacement once in two days (NR) | 1.PSQI; | The following are auricular points:  CO10 *Shen* 肾,  AT4 *Pizhixia* 皮质下,  TF4 *Shenmen* 神门,  CO13 *Pi* 脾,  AH6a *Jiaogan* 交感,  CO15 *Xin* 心, |
| **Liao et al. (23)**  **2018** | 36-70  /36-71Y | 12/17 | 11/18 | IN+OT | IN | NR | CCMD-2 | 8 times (4W)  /8 times (4W) | 1.PSQI; 2.effective rate; 3.AE | BL15 *Xinshu* 心俞  BL23 *Shenshu* 肾俞  BL18 *Ganshu* 肝俞  GB20 *Fengchi* 风池  HT7 *Shenmen* 神门  PC6 *Neiguan* 内关  ST36 *Zusanli* 足三里  SP6 *Sanyinjiao* 三阴交 |
| **Li et al. (48)**  **2020** | 67.6±7.6  /67.0±7.2Y | NR | NR | TEAS | PSA | IF, CCA, OC | NR | NR/NR | 1.PSQI; | PC6 *Neiguan* 内关  LI4 *Hegu* 合谷 |
| **Li et al. (47)**  **2020** | 56.67±13.11  /56.67±13.11Y | 20/21 | 20/19 | RC+AA | RC | NR | Guidelines for Clinical Research of New Chinese Medicines New Drug | 3 times (10D)  /NR (10D) | 1.PSQI; 2.effective rate | The following are auricular points:  CO15 *Xin* 心,  CO14 *Fei* 肺,  CO10 *Shen* 肾,  AT4 *Pizhixia* 皮质下,  CO12 *Gan* 肝,  CO13 *Pi* 脾,  CO4 *Wei* 胃,  CO7 *Dachang* 大肠,  AH6a *Jiaogan* 交感. |
| **Li et al. (36)**  **2019** | 35-67（46.65±7.43）  /33-65（45.01±6.28）Y | N | NR | WM  +AA | WM | BC | Guidelines for the diagnosis and treatment of adult insomnia in China and SDECMS | Press 3 times a day， replace once a day (2W)  /Once daily (2W) | 1.PSQI; 2.effective rate | The following are auricular points:  TF4 *Shenmen* 神门,  CO15 *Xin* 心,  AT4 *Pizhixia* 皮质下,  CO13 *Pi* 脾,  CO12 *Gan* 肝,  CO18 *Neifenmi* 内分泌,  AH6a *Jiaogan* 交感. |
| **Lu et al. (46)**  **2015** | 62.11±9.03  /62.11±8.57Y | 65/35 | 66/34 | AA+OT | WM | GCA, NPC, GBC, LC, LCA, HCC, CRC, PCa | NR | 5 times (15D)  /Once daily (7D) | 1.PSQI; | The following are auricular points:  TF4 *Shenmen* 神门,  CO15 *Xin* 心,  CO10 *Shen* 肾,  CO12 *Gan* 肝,  CO14 *Fei* 肺,  CO17 *Sanjiao* 三焦. |
| **Peng et al. (32)**  **2016** | 59.36±12.54  /60.95±11.11Y | 68/36 | 68/36 | ACU  +MOX | WM | NR | SDECMS | Once daily (1W)  /Once daily (1W) | 1.effective rate； | GV20 *Baihui* 百会  GV24 *Shenting* 神庭  GV29 *Yintang* 印堂  HT7 *Shenmen* 神门  ST36 *Zusanli* 足三里 |
| **Garland et al. (26)**  **2019** | 62.3/60.7Y | 37/43 | 32/48 | AA  +ACU | CBT | BC, PCa, CRC  etc. | DSM-5 | 10 times (8W)  /7 times (8W) | 1.PSQI; 2.AE | HT7 *Shenmen* 神门  SP6 *Sanyinjiao* 三阴交  GV20 *Baihui* 百会  GV24 *Shenting* 神庭 |
| **Shi et al. (25)**  **2020** | 49.5±5.72  /48.98±5.77Y | 54/46 | 58/42 | SA | ACU | NR | CCMD-3 | Once daily (1W)  /NR  . | 1.PSQI; 2.AE. | GV20 *Baihui* 百会  GV22 Xinhui 囟会  GV24 *Shenting* 神庭  GV29 *Yintang* 印堂  GB15 *Toulinqi* 头临泣  GB17 *Zhengying* 正营  EX-HN5 *Taiyang* 太阳 |
| **Shi (10)**  **2020** | 48.7±5.4  /44.9±5.1Y | 24/16 | 22/18 | SA | WM | NR | CCMD-3 | Once daily (3W)  /Once daily (3W) | 1.PSQI; 2.effective rate | GV20 *Baihui* 百会  GV22 Xinhui 囟会  GV24 *Shenting* 神庭  GV29 *Yintang* 印堂  GB15 *Toulinqi* 头临泣  GB17 *Zhengying* 正营  EX-HN5 *Taiyang* 太阳 |
| **Song et al. (30)**  **2015** | 18-80Y | NR | NR | ACU  +MOX | WM | NR | CCMD-3 | Once daily (1W)  /Once daily (1W) | 1. PSQI;   2. effective rate | GV20 *Baihui* 百会  GV24 *Shenting* 神庭  HT7 *Shenmen* 神门  GV29 *Yintang* 印堂  SP6 *Sanyinjiao* 三阴交  ST36 *Zusanli* 足三里  CV8 *Shenque* 神阙  CV4 *Guanyuan* 关元 |
| **Song et al. (44)**  **2014** | NR | NR | NR | RC+AA | RC | GC, LCA, HCC, GBC | NR | Replacement once in two days (10D)  /NR (10D) | 1.PSQI;  2. effective rate | The following are auricular points:  TF4 *Shenmen* 神门,  CO15 *Xin* 心,  AT4 *Pizhixia* 皮质下,  AH6a *Jiaogan* 交感,  CO12 *Gan* 肝,  CO14 *Fei* 肺,  CO11 Yi*Dan* 胰胆,  CO4 *Wei* 胃, |
| **Wang et al. (27)**  **2021** | 42.99±11.75  /42.96±11.95Y | NR | NR | RC  +MOX | WM | BC | DSM-5 | Once daily (14D)  /Once daily (14D) | 1.PSQI; 2.effective rate; 3AE | BL15 *Xinshu* 心俞  BL20 *Pishu* 脾俞  KI3 *Taixi* 太溪  GV20 *Baihui* 百会  KI1 *Yongquan*涌泉  HT7 *Shenmen* 神门 |
| **Wang et al. (12)**  **2019** | 52.47±9.62  /50.16±7.34Y | NR | NR | IN | PSA | BC | Guidelines for the diagnosis and treatment of adult insomnia in China | 8 times (4W)  /8 times (4W) | 1.PSQI; 2.effective rate | HT7 *Shenmen* 神门  SP6 *Sanyinjiao* 三阴交  EX-HN22 *Anmian* 安眠  ST36 *Zusanli* 足三里  SP3 *Taibai* 太白 |
| **Wang et al. (45)**  **2020** | 47±8  /48±8Y | 34/16 | 33/17 | TEAS | PSA | EC | NR | NR/NR | 1.PSQI; 2.AE | PC6 *Neiguan* 内关  LI4 *Hegu* 合谷 |
| **Xia et al. (40)**  **2019** | 47.6±8.20  /46.2±6.50Y | 10/27 | 13/24 | RC  +MOX | RC | TC | NR | Once daily (7D)  /NR (7D) | 1.PSQI; | GV20 *Baihui* 百会 |
| **Ye et al. (31)**  **2013** | ≥31/ ≥32Y | 24/17 | 22/18 | AA  +ACU | WM | LCA, BC, HCC, CRC, GC, OC, PC, RCC | CCMD-3 | 3 times a day (NR)  /Once daily (NR) | 1.effective rate | The following are auricular points:  TF4 *Shenmen* 神门,  CO15 *Xin* 心,  AT4 *Pizhixia* 皮质下,  AH6a *Jiaogan* 交感,  CO12 *Gan* 肝,  CO11 *Dan* 胆,  CO13 *Pi* 脾,  CO10 *Shen* 肾,  CO18 *Neifenmi* 内分泌,  CO17 *Sanjiao* 三焦,  CO6 *Xiaochang* 小肠,  HX4 *Waishengzhiqi*外生殖器,  TF2 *Neishengzhiqi*内生殖器. |
| **Yin et al. (37)**  **2021** | 54±8  /55±9Y | 26/19 | 24/21 | WM  +ACU  +MOX | WM | LCA, HCC, GC , EC. | ICSD-3、CCMD-3、ICD-10 | Once daily (2W)  /3 times a day (2W) | 1. PSQI;   2.effective rate;  3.AE | GV20 *Baihui* 百会  GV24 *Shenting* 神庭  GV29 *Yintang* 印堂  SP6 *Sanyinjiao* 三阴交  HT7 *Shenmen* 神门  ST36 *Zusanli* 足三里  CV8 *Shenque* 神阙  CV4 *Guanyuan* 关元 |
| **Yu et al. (41)**  **2018** | 31-77(52)  /36-78(53)Y | 23/22 | 28/29 | RC  +MOX | RC | LCA, BC, GC, OC ,CRC | Guidelines for Clinical Research of New Chinese Medicines New Drug | Once daily (14D)  /Once daily (14D) | 1.PSQI; 2.effective rate | SP6 *Sanyinjiao* 三阴交  KI1 *Yongquan*涌泉 |
| **Zhang (33)**  **2021** | 47.23±1.19  /48.13±1.22Y | 55/56 | 55/54 | WM  +AA  +MOX | WM  +AA | LCA | SDECMS | AA 15times (1M)  MOX (1M)  /AA 15 times (1M) | 1.effective rate | The following are auricular points:  TF4 *Shenmen* 神门,  CO15 *Xin* 心,  AT4 *Pizhixia* 皮质下,  *Shimian* 失眠,  AT3 *Zhen* 枕,  LO4 *Chuiqian* 垂前,  CO12 *Gan* 肝,  CO13 *Pi* 脾. |
| **Zhang et al. (24)**  **2022** | ＞18Y | N | 60 | RC  +AA  +MOX | RC  +AA | BC | NR | Once daily (3D)  /Press 3-5 times a day (NR) | 1.PSQI; 2.effective rate | BL15 *Xinshu* 心俞  BL23 *Shenshu* 肾俞  HT7 *Shenmen* 神门  GV29 *Yintang* 印堂  GV20 *Baihui* 百会  KI3 *Taixi* 太溪 |
| **Zhang et al. (42)**  **2022** | 53.85±8.93  /52.38±8.94Y | NR | NR | RC+IN | RC | BC | Guidelines for the diagnosis and treatment of adult insomnia in China | 8 times (4W)  /Once daily (14D) | 1.PSQI; 2.effective rate | HT7 *Shenmen* 神门  EX-HN22 *Anmian* 安眠  ST36 *Zusanli* 足三里  SP6 *Sanyinjiao* 三阴交  SP3 *Taibai* 太白 |
| **Zhang et al. (39)**  **2021** | 42.37±7.45  /43.16±7.87Y | N | NR | RC+AA | RC | CCA | NR | Press 4 times a day， replacement once in two days (4W)  /NR (4W) | 1. PSQI; | The following are auricular points:  TF4 *Shenmen* 神门,  CO15 *Xin* 心,  AT4 *Pizhixia* 皮质下,  AH6a *Jiaogan* 交感,  CO13 *Pi* 脾,  CO18 *Neifenmi* 内分泌,  CO6 *Xiaochang* 小肠,  CO12 *Gan* 肝,  CO11 *Dan* 胆,  CO10 *Shen* 肾,  AT3 *Zhen* 枕,  HX6,7 i *Erjian* 耳尖,  CO9 *Pangguang* 膀胱. |
| **Zhao (43)**  **2020** | 40-60Y | NR | NR | WM  +AA | WM | NR | NR | Press 4-6 times a day, replacement 1-2 days (NR)  /NR | 1.effective rate | NR |
| **Zheng (38)**  **2018** | 57.32±4.49  /58.38±5.31Y | 18/12 | 17/13 | RC+AA | RC | NR | NR | 3-5 times a day (8W)  /Once daily (8W) | 1.PSQI; | The following are auricular points:  TF4 *Shenmen* 神门,  CO15 *Xin* 心,  AT4 *Pizhixia* 皮质下,  CO13 *Pi* 脾,  CO10 *Shen* 肾, |
| **He et al. (34)**  **2016** | NR | 11/14 | 32/29 | RC  +MOX | RC | TC | ICD-10 | 1-2times a day (3D)  /1-2times a day (3D) | 1.PSQI; 2.effective rate | GV20 *Baihui* 百会 |
| **Lee (11)**  **2022** | 57.63 (52.53, 62.72)  /62.33 (54.43, 70.23)  /61.38 (55.12, 67.63) | 2/5/3 | 6/1/5 | EA | PSA  /RC | BC, TC, CRC | DSM-5 | 10 times (4W)  /10 times (4W) | 1.PSQI; | GV20 *Baihui* 百会  GV29 *Yintang* 印堂  HT7 *Shenmen* 神门  PC6 *Neiguan* 内关  BL63 *Jingmen* 京门  KI4 *Dazhong* 大钟 |

E, experimental group; C, control group; D, day; W, week; M, month; Y, year; NR, not reported; N, none; AA, auricular acupuncture; WM, western medicine; RC, routine care; AA, auricular acupuncture; CBT, cognitive behavioral therapy; ACU+MOX, acupuncture and moxibustion; AA+ACU: auricular acupuncture combined with acupuncture; RC+AA: routine care combined with auricular acupuncture; RC+MOX: routine care combined with moxibustion; RC+IN: routine care combined with intradermal needling; RC+AA+MOX: routine care combined with auricular acupuncture and moxibustion; WM+AA+MOX: western medicine combined with auricular acupuncture and moxibustion; WM+ACU+MOX: western medicine combined with acupuncture and moxibustion; WM+AA: western medicine combined with auricular acupuncture; SA: scalp-acupuncture; ACU: acupuncture; EA: electro-acupuncture; IN: intradermal needling; IN+OT: intradermal needling combined with other therapies; TEAS: transcutaneous electrical acupoint stimulation; AA+OT: acupuncture combined with other therapies; PSA: placebo-sham acupuncture. BC, breast cancer; PCa, prostate cancer; LCA, lung cancer; HCC, hepatocellular carcinoma; GC, gastric cancer; EC, esophagus cancer; CRC, colorectal cancer; LC, lymph cancer; CCA, cervical cancer; OC, ovarian cancer; PC, pancreatic cancer; RCC, renal cell carcinoma; GBC, gallbladder cancer; CWM, conventional western medicine; TC, thyroid cancer; GCA, gastric cardia adenocarcinoma; NPC, nasopharyngeal carcinoma; UF, uterine fibroids; PSQI, Pittsburgh Sleeps Quality Index; AE, adverse events; CCMD-3, Chinese Classification of Mental Disorder, third version; DSM-5, the Diagnostic and Statistical Manual of Mental Disorders, fifth edition; SDECMS, Standard for Diagnosis and Efficacy of Chinese Medicine Syndrome; CCMD-2, Chinese Classification of Mental Disorder, second version; ICD-10, International Classification of Diseases, tenth version.

TABLE 3| Head-to-head comparisons for PSQI of the different interventions

| **TEAS** | **ACU+MOX** | **RC** | **WM** | **PSA** | **AA** | **RC+IN** | **IN** | **SA** | **RC+AA** | **RC+MOX** | **CBT** | **AA+ACU** | **IN+OT** | **EA** | **WM+AA** | **AA+OT** | **ACU** | **RC+AA+MOX** | **WM+ACU+MOX** |
| --- | --- | --- | --- | --- | --- | --- | --- | --- | --- | --- | --- | --- | --- | --- | --- | --- | --- | --- | --- |
| **TEAS** | 1.32 (-7.96,10.60) | 1.95 (-5.16,9.05) | -1.81 (-11.21,7.60) | 2.19 (0.03,4.34) | 3.05 (-5.18,11.27) | 3.81 (-4.34,11.95) | 3.86 (-1.31,9.02) | 4.37 (-4.89,13.63) | 4.63 (-2.72,11.99) | 4.79 (-2.68,12.26) | 5.14 (-5.16,15.44) | 5.31 (-4.25,14.87) | 5.82 (-0.69,12.32) | 5.58 (0.36,10.79) | 6.04 (-3.27,15.35) | 6.07 (-3.18,15.32) | 6.13 (-3.12,15.38) | 6.60 (-1.70,14.91) | 6.97 (-2.29,16.23) |
| -1.32 (-10.60,7.96) | **ACU+MOX** | 0.63 (-5.34,6.59) | 0.73 (-3.14,4.60) | 0.87 (-8.16,9.89) | 1.73 (-5.53,8.98) | 2.49 (-4.69,9.66) | 2.54 (-7.63,12.71) | 3.05 (-2.39,8.49) | 3.32 (-2.95,9.58) | 3.47 (-2.04,8.98) | 3.82 (-3.25,10.89) | 3.99 (-1.94,9.92) | 4.50 (-6.42,15.41) | 4.26 (-3.42,11.93) | 4.72 (-0.81,10.25) | 4.75 (-0.68,10.18) | 4.81 (-0.61,10.23) | 5.29 (-2.06,12.64) | 5.65 (0.21,11.09) |
| -1.95 (-9.05,5.16) | -0.63 (-6.59,5.34) | **RC** | 0.10 (-4.44,4.64) | 0.24 (-6.53,7.01) | 1.10 (-3.04,5.24) | 1.86 (-2.12,5.84) | 1.91 (-6.33,10.15) | 2.42 (-3.51,8.36) | 2.69 (0.79,4.59) | 2.84 (0.56,5.13) | 3.19 (-4.26,10.65) | 3.36 (-3.03,9.75) | 3.87 (-5.27,13.01) | 3.63 (-1.20,8.46) | 4.09 (-1.92,10.11) | 4.12 (-1.80,10.05) | 4.18 (-1.74,10.10) | 4.66 (0.36,8.95) | 5.02 (-0.91,10.96) |
| 1.81 (-7.60,11.21) | -0.73 (-4.60,3.14) | -0.10 (-4.64,4.44) | **WM** | -2.05 (-10.48,6.38) | 1.00 (-5.14,7.14) | 2.74 (-1.18,6.66) | 4.08 (0.28,7.88) | 3.99 (0.04,7.94) | 0.41 (-5.49,6.31) | 2.59 (-2.34,7.51) | 3.09 (-2.82,9.00) | 3.26 (-1.24,7.76) | 3.53 (-3.10,10.15) | -2.06 (-8.62,4.50) | 4.92 (1.09,8.75) | 3.77 (-6.44,13.97) | 2.32 (-1.50,6.14) | 1.76 (-4.28,7.80) | 4.56 (-1.69,10.80) |
| ***-2.19 (-4.34,-0.03)*** | -0.87 (-9.89,8.16) | -0.24 (-7.01,6.53) | 2.05 (-6.38,10.48) | **PSA** | 0.86 (-7.08,8.80) | 1.62 (-6.24,9.48) | 1.67 (-3.02,6.36) | 2.18 (-6.82,11.19) | 2.45 (-4.59,9.49) | 2.60 (-4.55,9.75) | 2.95 (-7.12,13.02) | 3.12 (-6.19,12.43) | 3.63 (-2.51,9.77) | 3.39 (-1.36,8.14) | 3.85 (-5.20,12.91) | 3.88 (-5.11,12.88) | 3.94 (-5.05,12.94) | 4.42 (-3.60,12.44) | 4.78 (-4.22,13.79) |
| -3.05 (-11.27,5.18) | -1.73 (-8.98,5.53) | -1.10 (-5.24,3.04) | -1.00 (-7.14,5.14) | -0.86 (-8.80,7.08) | **AA** | 0.76 (-4.98,6.50) | 0.81 (-8.41,10.03) | 1.32 (-5.91,8.55) | 1.59 (-2.96,6.14) | 1.74 (-2.98,6.47) | 2.09 (-6.43,10.62) | 2.26 (-5.35,9.88) | 2.77 (-7.26,12.80) | 2.53 (-3.83,8.89) | 2.99 (-4.30,10.29) | 3.02 (-4.20,10.25) | 3.08 (-4.14,10.31) | 3.56 (-2.40,9.52) | 3.92 (-3.31,11.16) |
| -3.81 (-11.95,4.34) | -2.49 (-9.66,4.69) | -1.86 (-5.84,2.12) | -2.74 (-6.66,1.18) | -1.62 (-9.48,6.24) | -0.76 (-6.50,4.98) | **RC+IN** | 0.05 (-9.10,9.20) | 0.56 (-6.58,7.71) | 0.83 (-3.59,5.24) | 0.98 (-3.61,5.58) | 1.33 (-7.12,9.78) | 1.50 (-6.03,9.03) | 2.01 (-7.96,11.98) | 1.77 (-4.49,8.03) | 2.23 (-4.98,9.45) | 2.26 (-4.87,9.40) | 2.32 (-4.81,9.46) | 2.80 (-3.06,8.66) | 3.16 (-3.98,10.31) |
| -3.86 (-9.02,1.31) | -2.54 (-12.71,7.63) | -1.91 (-10.15,6.33) | ***-4.08 (-7.88,-0.28)*** | -1.67 (-6.36,3.02) | -0.81 (-10.03,8.41) | -0.05 (-9.20,9.10) | **IN** | 0.51 (-9.64,10.67) | 0.78 (-7.68,9.24) | 0.93 (-7.62,9.48) | 1.28 (-9.83,12.39) | 1.45 (-8.97,11.88) | 1.96 (-2.00,5.92) | 1.72 (-4.96,8.40) | 2.18 (-8.02,12.38) | 2.21 (-7.93,12.36) | 2.27 (-7.87,12.42) | 2.75 (-6.54,12.04) | 3.11 (-7.04,13.27) |
| -4.37 (-13.63,4.89) | -3.05 (-8.49,2.39) | -2.42 (-8.36,3.51) | ***-3.99 (-7.94,-0.04)*** | -2.18 (-11.19,6.82) | -1.32 (-8.55,5.91) | -0.56 (-7.71,6.58) | -0.51 (-10.67,9.64) | **SA** | 0.27 (-5.96,6.50) | 0.42 (-5.05,5.89) | 0.77 (-6.27,7.81) | 0.94 (-4.96,6.84) | 1.45 (-9.45,12.34) | 1.21 (-6.44,8.86) | 1.67 (-3.82,7.16) | 1.70 (-3.69,7.09) | 1.76 (-3.63,7.15) | 2.24 (-5.09,9.56) | 2.60 (-2.80,8.01) |
| -4.63 (-11.99,2.72) | -3.32 (-9.58,2.95) | ***-2.69 (-4.59,-0.79)*** | -0.41 (-6.31,5.49) | -2.45 (-9.49,4.59) | -1.59 (-6.14,2.96) | -0.83 (-5.24,3.59) | -0.78 (-9.24,7.68) | -0.27 (-6.50,5.96) | **RC+AA** | 0.15 (-2.82,3.13) | 0.50 (-7.19,8.20) | 0.67 (-5.99,7.34) | 1.18 (-8.16,10.52) | 0.94 (-4.25,6.13) | 1.40 (-4.90,7.71) | 1.43 (-4.79,7.66) | 1.49 (-4.72,7.71) | 1.97 (-1.88,5.82) | 2.33 (-3.90,8.57) |
| -4.79 (-12.26,2.68) | -3.47 (-8.98,2.04) | ***-2.84 (-5.13,-0.56)*** | -2.59 (-7.51,2.34) | -2.60 (-9.75,4.55) | -1.74 (-6.47,2.98) | -0.98 (-5.58,3.61) | -0.93 (-9.48,7.62) | -0.42 (-5.89,5.05) | -0.15 (-3.13,2.82) | **RC+MOX** | 0.35 (-6.74,7.44) | 0.52 (-5.45,6.49) | 1.03 (-8.40,10.45) | 0.79 (-4.56,6.13) | 1.25 (-4.31,6.81) | 1.28 (-4.18,6.74) | 1.34 (-4.12,6.80) | 1.82 (-3.05,6.68) | 2.18 (-3.30,7.66) |
| -5.14 (-15.44,5.16) | -3.82 (-10.89,3.25) | -3.19 (-10.65,4.26) | -3.09 (-9.00,2.82) | -2.95 (-13.02,7.12) | -2.09 (-10.62,6.43) | -1.33 (-9.78,7.12) | -1.28 (-12.39,9.83) | -0.77 (-7.81,6.27) | -0.50 (-8.20,7.19) | -0.35 (-7.44,6.74) | **CBT** | 0.17 (-3.67,4.01) | 0.68 (-11.12,12.47) | 0.44 (-8.45,9.32) | 0.90 (-6.21,8.01) | 0.93 (-6.10,7.96) | 0.99 (-6.04,8.02) | 1.47 (-7.14,10.07) | 1.83 (-5.21,8.87) |
| -5.31 (-14.87,4.25) | -3.99 (-9.92,1.94) | -3.36 (-9.75,3.03) | -3.26 (-7.76,1.24) | -3.12 (-12.43,6.19) | -2.26 (-9.88,5.35) | -1.50 (-9.03,6.03) | -1.45 (-11.88,8.97) | -0.94 (-6.84,4.96) | -0.67 (-7.34,5.99) | -0.52 (-6.49,5.45) | -0.17 (-4.01,3.67) | **AA+ACU** | 0.51 (-10.65,11.66) | 0.27 (-7.74,8.28) | 0.73 (-5.25,6.71) | 0.76 (-5.13,6.65) | 0.82 (-5.07,6.71) | 1.30 (-6.40,9.00) | 1.66 (-4.25,7.57) |
| -5.82 (-12.32,0.69) | -4.50 (-15.41,6.42) | -3.87 (-13.01,5.27) | -3.53 (-10.15,3.10) | -3.63 (-9.77,2.51) | -2.77 (-12.80,7.26) | -2.01 (-11.98,7.96) | -1.96 (-5.92,2.00) | -1.45 (-12.34,9.45) | -1.18 (-10.52,8.16) | -1.03 (-10.45,8.40) | -0.68 (-12.47,11.12) | -0.51 (-11.66,10.65) | **IN+OT** | -0.24 (-8.00,7.52) | 0.22 (-10.72,11.16) | 0.25 (-10.64,11.14) | 0.31 (-10.57,11.20) | 0.79 (-9.31,10.89) | 1.15 (-9.74,12.05) |
| ***-5.58 (-10.79,-0.36)*** | -4.26 (-11.93,3.42) | -3.63 (-8.46,1.20) | 2.06 (-4.50,8.62) | -3.39 (-8.14,1.36) | -2.53 (-8.89,3.83) | -1.77 (-8.03,4.49) | -1.72 (-8.40,4.96) | -1.21 (-8.86,6.44) | -0.94 (-6.13,4.25) | -0.79 (-6.13,4.56) | -0.44 (-9.32,8.45) | -0.27 (-8.28,7.74) | 0.24 (-7.52,8.00) | **EA** | 0.46 (-7.25,8.18) | 0.49 (-7.15,8.14) | 0.55 (-7.09,8.19) | 1.03 (-5.44,7.49) | 1.39 (-6.26,9.05) |
| -6.04 (-15.35,3.27) | -4.72 (-10.25,0.81) | -4.09 (-10.11,1.92) | ***-4.92 (-8.75,-1.09)*** | -3.85 (-12.91,5.20) | -2.99 (-10.29,4.30) | -2.23 (-9.45,4.98) | -2.18 (-12.38,8.02) | -1.67 (-7.16,3.82) | -1.40 (-7.71,4.90) | -1.25 (-6.81,4.31) | -0.90 (-8.01,6.21) | -0.73 (-6.71,5.25) | -0.22 (-11.16,10.72) | -0.46 (-8.18,7.25) | **WM+AA** | 0.03 (-5.45,5.51) | 0.09 (-5.39,5.57) | 0.57 (-6.83,7.96) | 0.93 (-4.57,6.43) |
| -6.07 (-15.32,3.18) | -4.75 (-10.18,0.68) | -4.12 (-10.05,1.80) | -3.77 (-13.97,6.44) | -3.88 (-12.88,5.11) | -3.02 (-10.25,4.20) | -2.26 (-9.40,4.87) | -2.21 (-12.36,7.93) | -1.70 (-7.09,3.69) | -1.43 (-7.66,4.79) | -1.28 (-6.74,4.18) | -0.93 (-7.96,6.10) | -0.76 (-6.65,5.13) | -0.25 (-11.14,10.64) | -0.49 (-8.14,7.15) | -0.03 (-5.51,5.45) | **AA+OT** | 0.06 (-5.32,5.44) | 0.54 (-6.78,7.85) | 0.90 (-4.50,6.30) |
| -6.13 (-15.38,3.12) | -4.81 (-10.23,0.61) | -4.18 (-10.10,1.74) | -2.32 (-6.14,1.50) | -3.94 (-12.94,5.05) | -3.08 (-10.31,4.14) | -2.32 (-9.46,4.81) | -2.27 (-12.42,7.87) | -1.76 (-7.15,3.63) | -1.49 (-7.71,4.72) | -1.34 (-6.80,4.12) | -0.99 (-8.02,6.04) | -0.82 (-6.71,5.07) | -0.31 (-11.20,10.57) | -0.55 (-8.19,7.09) | -0.09 (-5.57,5.39) | -0.06 (-5.44,5.32) | **ACU** | 0.48 (-6.84,7.79) | 0.84 (-4.55,6.23) |
| -6.60 (-14.91,1.70) | -5.29 (-12.64,2.06) | ***-4.66 (-8.95,-0.36)*** | -1.76 (-7.80,4.28) | -4.42 (-12.44,3.60) | -3.56 (-9.52,2.40) | -2.80 (-8.66,3.06) | -2.75 (-12.04,6.54) | -2.24 (-9.56,5.09) | -1.97 (-5.82,1.88) | -1.82 (-6.68,3.05) | -1.47 (-10.07,7.14) | -1.30 (-9.00,6.40) | -0.79 (-10.89,9.31) | -1.03 (-7.49,5.44) | -0.57 (-7.96,6.83) | -0.54 (-7.85,6.78) | -0.48 (-7.79,6.84) | **RC+AA+MOX** | 0.36 (-6.96,7.69) |
| -6.97 (-16.23,2.29) | -5.65 (-11.09,-0.21) | -5.02 (-10.96,0.91) | -4.56 (-10.80,1.69) | -4.78 (-13.79,4.22) | -3.92 (-11.16,3.31) | -3.16 (-10.31,3.98) | -3.11 (-13.27,7.04) | -2.60 (-8.01,2.80) | -2.33 (-8.57,3.90) | -2.18 (-7.66,3.30) | -1.83 (-8.87,5.21) | -1.66 (-7.57,4.25) | -1.15 (-12.05,9.74) | -1.39 (-9.05,6.26) | -0.93 (-6.43,4.57) | -0.90 (-6.30,4.50) | -0.84 (-6.23,4.55) | -0.36 (-7.69,6.96) | **WM+ACU+MOX** |

Mean difference and 95% credibility intervals between 17 interventions: indirect comparisons from network meta-analysis. Results are the MD and related 95% CIs in the row-defining treatment compared with the MD in the column-defining treatment. MD >0 favors the column-defining treatment and vice versa. The significant result is in bold and underlined.

WM, western medicine; RC, routine care; AA, auricular acupuncture; CBT, cognitive behavioral therapy; ACU+MOX, acupuncture and moxibustion; AA+ACU: auricular acupuncture combined with acupuncture; RC+AA: routine care combined with auricular acupuncture; RC+MOX: routine care combined with moxibustion; RC+IN: routine care combined with intradermal needling; RC+AA+MOX: routine care combined with auricular acupuncture and moxibustion; WM+ACU+MOX: western medicine combined with acupuncture and moxibustion; WM+AA: western medicine combined with auricular acupuncture; SA: scalp-acupuncture; ACU: acupuncture; EA: electro-acupuncture; IN: intradermal needling; IN+OT: intradermal needling combined with other therapies; TEAS: transcutaneous electrical acupoint stimulation; AA+OT: acupuncture combined with other therapies; PSA: placebo-sham acupuncture.

TABLE 4| Head-to-head comparisons for an effective rate of different interventions

| **RC+AA+MOX** | **WM+AA+MOX** | **RC+AA** | **RC+MOX** | **RC+IN** | **AA+ACU** | **WM+AA** | **SA** | **WM+ACU+MOX** | **RC** | **WM** | **ACU+MOX** |
| --- | --- | --- | --- | --- | --- | --- | --- | --- | --- | --- | --- |
| **RC+AA+MOX** | 0.91 (0.05,15.18) | 0.12 (0.02,0.62) | 0.10 (0.01,0.72) | 0.07 (0.01,0.53) | 0.06 (0.00,0.67) | 0.05 (0.00,0.61) | 0.04 (0.00,0.51) | 0.04 (0.00,0.51) | 0.02 (0.00,0.10) | 0.01 (0.00,0.09) | 0.00 (0.00,0.04) |
| 1.10 (0.07,18.48) | **WM+AA+MOX** | 0.14 (0.01,1.37) | 0.11 (0.01,0.75) | 0.08 (0.01,0.78) | 0.06 (0.01,0.44) | 0.05 (0.02,0.18) | 0.04 (0.01,0.34) | 0.04 (0.00,0.35) | 0.02 (0.00,0.16) | 0.01 (0.00,0.05) | 0.00 (0.00,0.03) |
| ***8.11 (1.61,40.77)*** | 7.35 (0.73,73.99) | **RC+AA** | 0.78 (0.23,2.61) | 0.56 (0.17,1.91) | 0.46 (0.07,2.98) | 0.39 (0.05,2.79) | 0.32 (0.04,2.35) | 0.29 (0.04,2.37) | 0.14 (0.06,0.32) | 0.07 (0.01,0.36) | 0.03 (0.01,0.19) |
| ***10.46 (1.39,78.88)*** | ***9.48 (1.33,67.59)*** | 1.29 (0.38,4.34) | **RC+MOX** | 0.73 (0.21,2.48) | 0.60 (0.15,2.45) | 0.50 (0.11,2.36) | 0.41 (0.08,2.01) | 0.38 (0.07,2.07) | 0.17 (0.07,0.41) | 0.09 (0.03,0.26) | 0.04 (0.01,0.15) |
| ***14.37 (1.90,108.76)*** | ***13.03 (1.29,131.96)*** | 1.77 (0.52,6.00) | 1.37 (0.40,4.68) | **RC+IN** | 0.82 (0.13,5.32) | 0.69 (0.10,4.97) | 0.56 (0.07,4.19) | 0.52 (0.06,4.22) | 0.24 (0.10,0.57) | 0.13 (0.03,0.64) | 0.06 (0.01,0.34) |
| ***17.46 (1.49,204.96)*** | ***15.83 (2.28,110.00)*** | 2.15 (0.34,13.83) | 1.67 (0.41,6.82) | 1.22 (0.19,7.85) | **AA+ACU** | 0.84 (0.19,3.82) | 0.68 (0.14,3.25) | 0.63 (0.12,3.36) | 0.29 (0.06,1.52) | 0.16 (0.06,0.41) | 0.07 (0.02,0.24) |
| ***20.76 (1.63,264.35)*** | ***18.82 (5.60,63.30)*** | 2.56 (0.36,18.29) | 1.99 (0.42,9.31) | 1.44 (0.20,10.38) | 1.19 (0.26,5.39) | **WM+AA** | 0.81 (0.15,4.38) | 0.75 (0.12,4.49) | 0.35 (0.06,2.04) | 0.19 (0.06,0.60) | 0.09 (0.02,0.33) |
| ***25.63 (1.95,336.58)*** | ***23.24 (2.91,185.85)*** | 3.16 (0.43,23.49) | 2.45 (0.50,12.09) | 1.78 (0.24,13.34) | 1.47 (0.31,7.01) | 1.23 (0.23,6.68) | **SA** | 0.92 (0.15,5.79) | 0.43 (0.07,2.63) | 0.23 (0.07,0.79) | 0.11 (0.03,0.43) |
| ***27.77 (1.97,390.74)*** | ***25.18 (2.89,219.22)*** | 3.43 (0.42,27.79) | 2.66 (0.48,14.61) | 1.93 (0.24,15.78) | 1.59 (0.30,8.49) | 1.34 (0.22,8.03) | 1.08 (0.17,6.79) | **WM+ACU+MOX** | 0.46 (0.07,3.14) | 0.25 (0.06,0.98) | 0.12 (0.03,0.53) |
| ***59.82 (9.62,371.89)*** | ***54.23 (6.34,463.51)*** | 7.38 (3.14,17.34) | ***5.72 (2.41,13.56)*** | ***4.16 (1.74,9.94)*** | 3.43 (0.66,17.85) | 2.88 (0.49,16.91) | 2.33 (0.38,14.32) | 2.15 (0.32,14.56) | **RC** | 0.54 (0.14,2.05) | 0.25 (0.06,1.12) |
| ***111.08 (11.55,1068.66)*** | ***100.70 (18.79,539.60)*** | ***13.71 (2.81,66.95)*** | ***10.62 (3.83,29.48)*** | ***7.73 (1.57,38.08)*** | ***6.36 (2.41,16.77)*** | ***5.35 (1.68,17.08)*** | ***4.33 (1.27,14.78)*** | ***4.00 (1.02,15.68)*** | 1.86 (0.49,7.07) | **WM** | 0.47 (0.24,0.91) |
| ***238.42 (22.50,2526.13)*** | ***216.14 (35.49,1316.41)*** | ***29.42 (5.26,164.45)*** | ***22.80 (6.73,77.22)*** | ***16.59 (2.94,93.48)*** | ***13.65 (4.21,44.31)*** | ***11.48 (3.01,43.82)*** | ***9.30 (2.30,37.60)*** | ***8.59 (1.88,39.28)*** | 3.99 (0.89,17.76) | ***2.15 (1.10,4.19)*** | **ACU+MOX** |

Odds ratio and 95% credibility intervals between 8 different interventions: indirect comparisons from network meta-analysis. Results are the ORs and related 95% CIs in the row-defining treatment compared with the ORs in the column-defining treatment. ORs>1 favor the column-defining treatment and vice versa. The significant result is in bold and underlined.

WM, western medicine; RC, routine care; ACU+MOX, acupuncture and moxibustion; AA+ACU: auricular acupuncture combined with acupuncture; RC+AA: routine care combined with auricular acupuncture; RC+MOX: routine care combined with moxibustion; RC+IN: routine care combined with intradermal needling; RC+AA+MOX: routine care combined with auricular acupuncture and moxibustion; WM+AA+MOX: western medicine combined with auricular acupuncture and moxibustion; WM+ACU+MOX: western medicine combined with acupuncture and moxibustion; WM+AA: western medicine combined with auricular acupuncture; SA: scalp-acupuncture.

TABLE 5| Evidence Quality of PSQI Score

| **Comparison** | | **Direct evidence** | **Indirect evidence** | **Network meta-analysis** |
| --- | --- | --- | --- | --- |
| TEAS | WM | Low | Low | Low |
| TEAS | PSA | Moderate | Low | Moderate |
| TEAS | RC | - | Low | Low |
| TEAS | CBT | - | Very low | Very low |
| TEAS | ACU+MOX | - | Low | Low |
| TEAS | AA+ACU | - | Low | Low |
| TEAS | RC+AA | - | Low | Low |
| TEAS | RC+MOX | - | Low | Low |
| TEAS | RC+IN | - | Low | Low |
| TEAS | RC+AA+MOX | - | Low | Low |
| TEAS | WM+ACU+MOX | - | Very Low | Low |
| TEAS | WM+AA | - | Low | Low |
| TEAS | SA | - | Low | Low |
| TEAS | ACU | - | Low | Low |
| TEAS | EA | Moderate | low | low |
| TEAS | IN | - | Low | Low |
| TEAS | IN+OT | - | Low | Low |
| TEAS | AA | - | Low | Low |
| TEAS | AA+OT | - | Low | Low |
| ACU+MOX | WM | Moderate | Moderate | Moderate |
| ACU+MOX | PSA | - | Low | Low |
| ACU+MOX | RC | - | Low | Low |
| ACU+MOX | CBT | - | Very Low | Low |
| ACU+MOX | AA+ACU | - | Low | Low |
| ACU+MOX | RC+AA | - | Low | Low |
| ACU+MOX | RC+MOX | - | Low | Low |
| ACU+MOX | RC+IN | - | Low | Low |
| ACU+MOX | RC+AA+MOX | - | Low | Low |
| ACU+MOX | WM+ACU+MOX | - | Low | Low |
| ACU+MOX | WM+AA | - | Low | Low |
| ACU+MOX | SA | - | Low | Low |
| ACU+MOX | ACU | - | Low | Low |
| ACU+MOX | EA | - | Very Low | Very Low |
| ACU+MOX | IN | - | Low | Low |
| ACU+MOX | IN+OT | - | Low | Low |
| ACU+MOX | AA | - | Low | Low |
| AA | WM | - | Low | Low |
| AA | PSA | - | Low | Low |
| AA | RC | Moderate | Low | Low |
| AA | CBT | - | Low | Low |
| AA | AA+ACU | - | Low | Low |
| AA | RC+AA | Low | Low | Very Low |
| AA | RC+MOX | Low | Low | Low |
| AA | RC+IN | - | Low | Low |
| AA | RC+AA+MOX | - | Low | Low |
| AA | WM+ACU+MOX | - | - | - |
| AA | WM+AA | - | Low | Low |
| AA | SA | - | Low | Low |
| AA | ACU | - | Low | Low |
| AA | EA | - | Low | Low |
| AA | IN | - | - | - |
| AA | IN+OT | - | Low | Low |
| AA | AA+OT | - | Very Low | Very Low |
| RC+IN | WM | - | Low | Low |
| RC+IN | PSA | - | Very Low | Very Low |
| RC+IN | RC | Moderate | Low | Low |
| RC+IN | CBT | - | Low | Low |
| RC+IN | AA+ACU | Low | Low | Low |
| RC+IN | RC+AA | - | Low | Low |
| RC+IN | RC+MOX | Low | Low | Low |
| RC+IN | RC+IN | - | Low | Low |
| RC+IN | RC+AA+MOX | - | Low | Low |
| RC+IN | WM+ACU+MOX | - | Low | Low |
| RC+IN | WM+AA | - | Very Low | Very Low |
| RC+IN | SA | - | - | - |
| RC+IN | ACU | - | Low | Low |
| RC+IN | EA | - | Low | Low |
| RC+IN | IN | - | Low | Low |
| RC+IN | IN+OT | - | Low | Low |
| RC+IN | AA+OT | - | Low | Low |
| IN | WM | - | Low | Low |
| IN | PSA | Moderate | Low | Low |
| IN | RC | - | Low | Low |
| IN | CBT | - | Low | Low |
| IN | AA+ACU | - | Low | Low |
| IN | RC+AA | - | Low | Low |
| IN | RC+MOX | - | Low | Low |
| IN | RC+AA+MOX | - | Low | Low |
| IN | WM+ACU+MOX | - | Low | Low |
| IN | WM+AA | - | Low | Low |
| IN | SA | - | Low | Low |
| IN | ACU | - | Low | Low |
| IN | EA | - | Low | Low |
| IN | IN | - | Low | Low |
| IN | IN+OT | Low | Low | Low |
| IN | AA+OT | - | Low | Low |
| SA | WM | Moderate | Low | Low |
| SA | PSA | - | Low | Low |
| SA | RC | - | Low | Low |
| SA | CBT | - | Low | Low |
| SA | AA+ACU | - | Low | Low |
| SA | RC+AA | - | Low | Low |
| SA | RC+MOX | - | Low | Low |
| SA | RC+AA+MOX | - | Very Low | Very Low |
| SA | WM+ACU+MOX | - | Low | Low |
| SA | WM+AA | - | Low | Low |
| SA | ACU | Low | Low | Low |
| SA | EA | - | Low | Low |
| SA | IN+OT | - | Low | Low |
| SA | AA+OT | - | Low | Low |
| AA+ACU | WM | - | Low | Low |
| AA+ACU | PSA | - | Low | Low |
| AA+ACU | RC | - | Low | Low |
| AA+ACU | CBT | Very low | Low | Very low |
| AA+ACU | RC+AA+MOX | - | Low | Low |
| AA+ACU | WM+ACU+MOX | - | Low | Low |
| AA+ACU | WM+AA | - | Low | Low |
| AA+ACU | ACU | - | Low | Low |
| AA+ACU | EA | - | Low | Low |
| AA+ACU | IN+OT | - | Low | Low |
| AA+ACU | AA+OT | - | Low | Low |
| IN+OT | WM | - | Low | Low |
| IN+OT | PSA | - | Low | Low |
| IN+OT | RC | - | Low | Low |
| IN+OT | CBT | - | Low | Low |
| IN+OT | RC+AA+MOX | - | Low | Low |
| IN+OT | WM+ACU+MOX | - | Low | Low |
| IN+OT | WM+AA | - | Low | Low |
| IN+OT | ACU | - | Low | Low |
| IN+OT | EA | - | Low | Low |
| IN+OT | IN+OT | - | Low | Low |
| IN+OT | AA+OT | - | Low | Low |
| EA | WM | - | - | - |
| EA | PSA | low | low | low |
| EA | RC | low | low | low |
| EA | CBT | - | low | low |
| EA | RC+AA+MOX | - | low | low |
| EA | WM+ACU+MOX | - | low | low |
| EA | WM+AA | - | low | low |
| EA | ACU | - | low | low |
| EA | IN+OT | - | low | low |
| EA | AA+OT | - | low | low |
| WM+AA | WM | low | low | low |
| WM+AA | PSA | - | low | low |
| WM+AA | RC | - | low | low |
| WM+AA | CBT | - | low | low |
| WM+AA | RC+AA+MOX | - | low | low |
| WM+AA | WM+ACU+MOX | - | low | low |
| WM+AA | ACU | - | low | low |
| WM+AA | IN+OT | - | low | low |
| WM+AA | AA+OT | - | low | low |
| AA+OT | WM | low | low | low |
| AA+OT | PSA | - | low | low |
| AA+OT | RC | - | low | low |
| AA+OT | CBT | - | low | low |
| AA+OT | RC+AA+MOX | - | low | low |
| AA+OT | WM+ACU+MOX | - | low | low |
| AA+OT | ACU | - | low | low |
| ACU | WM | low | low | low |
| ACU | PSA | - | low | low |
| ACU | RC | - | low | low |
| ACU | CBT | - | low | low |
| ACU | RC+AA+MOX | - | low | low |
| ACU | WM+ACU+MOX | - | low | low |
| RC+AA+MOX | WM | - | low | low |
| RC+AA+MOX | PSA | - | low | low |
| RC+AA+MOX | RC | - | low | low |
| RC+AA+MOX | CBT | - | low | low |
| RC+AA+MOX | WM+AA+MOX | - | low | low |
| WM+ACU+MOX | WM | - | low | low |
| WM+ACU+MOX | PSA | - | low | low |
| WM+ACU+MOX | RC | - | low | low |
| WM+ACU+MOX | CBT | - | low | low |
| RC+MOX | WM | - | low | low |
| RC+MOX | PSA | - | low | low |
| RC+MOX | RC | low | low | low |
| RC+MOX | CBT | - | low | low |
| RC+MOX | AA+ACU | - | low | low |
| RC+MOX | RC+AA+MOX | - | low | low |
| RC+MOX | WM+ACU+MOX | - | low | low |
| RC+MOX | WM+AA | - | low | low |
| RC+MOX | ACU | - | low | low |
| RC+MOX | EA | - | low | low |
| RC+MOX | IN+OT | - | low | low |
| RC+AA | WM | low | low | low |
| RC+AA | PSA | - | low | low |
| RC+AA | RC | - | low | low |
| RC+AA | CBT | - | low | low |
| RC+AA | AA+ACU | - | low | low |
| RC+AA | RC+MOX | - | low | low |
| RC+AA | RC+AA+MOX | - | low | low |
| RC+AA | WM+ACU+MOX | - | low | low |
| RC+AA | WM+AA | - | low | low |
| RC+AA | ACU | - | low | low |
| RC+AA | EA | - | low | low |
| RC+AA | IN+OT | - | low | low |

WM, western medicine; RC, routine care; AA, auricular acupuncture; CBT, cognitive behavioral therapy; ACU+MOX, acupuncture and moxibustion; AA+ACU: auricular acupuncture combined with acupuncture; RC+AA: routine care combined with auricular acupuncture; RC+MOX: routine care combined with moxibustion; RC+IN: routine care combined with intradermal needling; RC+AA+MOX: routine care combined with auricular acupuncture and moxibustion; WM+ACU+MOX: western medicine combined with acupuncture and moxibustion; WM+AA: western medicine combined with auricular acupuncture; SA: scalp-acupuncture; ACU: acupuncture; EA: electro-acupuncture; IN: intradermal needling; IN+OT: intradermal needling combined with other therapies; TEAS: transcutaneous electrical acupoint stimulation; AA+OT: acupuncture combined with other therapies; PSA: placebo-sham acupuncture.

TABLE 6| Evidence Quality of  effective rate

| **Comparison** | | **Direct evidence** | **Indirect evidence** | **Network meta-analysis** |
| --- | --- | --- | --- | --- |
| RC+AA+MOX | WM+AA+MOX | - | Low | Low |
| RC+AA+MOX | RC+AA | Moderate | Moderate | Moderate |
| RC+AA+MOX | RC+MOX | - | Low | Low |
| RC+AA+MOX | RC+IN | - | low | Very low |
| RC+AA+MOX | AA+ACU | - | Very low | Very low |
| RC+AA+MOX | WM+AA | - | Low | Low |
| RC+AA+MOX | SA | - | Low | Low |
| RC+AA+MOX | WM+ACU+MOX | - | Low | Low |
| RC+AA+MOX | RC | - | Low | Low |
| RC+AA+MOX | WM | - | Low | Low |
| RC+AA+MOX | ACU+MOX | Low | Low | Low |
| RC+AA | WM+AA+MOX | - | Low | Low |
| RC+AA | RC+MOX | - | Very low | Very low |
| RC+AA | RC+IN | - | Low | Low |
| RC+AA | AA+ACU | Low | Low | Low |
| RC+AA | WM+AA | - | Low | Low |
| RC+AA | SA | - | Low | Low |
| RC+AA | WM+ACU+MOX | - | Low | Low |
| RC+AA | RC | Low | Low | Low |
| RC+AA | WM | Low | Low | Low |
| RC+AA | ACU+MOX | - | Low | Low |
| RC+MOX | RC+IN | - | Very Low | Very Low |
| RC+MOX | AA+ACU | - | Low | Low |
| RC+MOX | WM+AA | - | Low | Low |
| RC+MOX | SA | - | Low | Low |
| RC+MOX | WM+ACU+MOX | - | Low | Low |
| RC+MOX | RC | - | Low | Low |
| RC+MOX | WM | Low | Low | Low |
| RC+MOX | ACU+MOX | - | Low | Low |
| RC+IN | AA+ACU | - | Low | Low |
| RC+IN | WM+AA | - | Low | Low |
| RC+IN | SA | - | Low | Low |
| RC+IN | WM+ACU+MOX | - | Low | Low |
| RC+IN | RC | Low | Low | Low |
| RC+IN | WM | - | Low | Low |
| RC+IN | AA | - | Low | Low |
| AA+ACU | WM+AA | - | Low | Low |
| AA+ACU | SA | - | Low | Low |
| AA+ACU | WM+ACU+MOX | - | Low | Low |
| AA+ACU | RC | - | Low | Low |
| AA+ACU | WM | Low | Low | Low |
| AA+ACU | ACU+MOX | - | Low | Low |
| WM+AA | SA | - | Low | Low |
| WM+AA | WM+ACU+MOX | - | Low | Low |
| WM+AA | RC | - | Low | Low |
| WM+AA | WM | Low | Low | Low |
| WM+AA | AA | - | Low | Low |
| SA | WM+ACU+MOX | - | Low | Low |
| SA | RC | - | Low | Low |
| SA | WM | Low | Low | Low |
| SA | ACU+MOX | - | Low | Low |
| WM+ACU+MOX | RC | - | Low | Low |
| WM+ACU+MOX | WM | - | Low | Low |
| WM+ACU+MOX | ACU+MOX | - | Low | Low |
| ACU+MOX | RC | - | Low | Low |
| ACU+MOX | WM | - | Low | Low |
| WM+AA+MOX | RC+AA | - | Low | Low |
| WM+AA+MOX | RC+MOX | - | Low | Low |
| WM+AA+MOX | RC+IN | - | Low | Low |
| WM+AA+MOX | AA+ACU | - | Low | Low |
| WM+AA+MOX | WM+AA | Moderate | Low | Low |
| WM+AA+MOX | SA | - | Low | Low |
| WM+AA+MOX | WM+ACU+MOX | - | Low | Low |
| WM+AA+MOX | RC | - | Low | Low |
| WM+AA+MOX | WM | - | Low | Low |
| WM+AA+MOX | ACU+MOX | - | Low | Low |

WM, western medicine; RC, routine care; ACU+MOX, acupuncture and moxibustion; AA+ACU: auricular acupuncture combined with acupuncture; RC+AA: routine care combined with auricular acupuncture; RC+MOX: routine care combined with moxibustion; RC+IN: routine care combined with intradermal needling; RC+AA+MOX: routine care combined with auricular acupuncture and moxibustion; WM+AA+MOX: western medicine combined with auricular acupuncture and moxibustion; WM+ACU+MOX: western medicine combined with acupuncture and moxibustion; WM+AA: western medicine combined with auricular acupuncture; SA: scalp-acupuncture.
